# Polarized Laser Switching with Giant Contrast in MOF‐Based Mixed‐Matrix Membrane

**DOI:** 10.1002/advs.202200953

**Published:** 2022-04-11

**Authors:** Hongjun Li, Lin Zhang, Yu Yang, Enlai Hu, Bin Li, Yuanjing Cui, Deren Yang, Guodong Qian

**Affiliations:** ^1^ State Key Laboratory of Silicon Materials Cyrus Tang Center for Sensor Materials and Applications School of Materials Science and Engineering Zhejiang University Hangzhou 310027 China

**Keywords:** metal‐organic framework, mixed‐matrix membranes, nonlinear optical switch, two‐photon pumped lasing

## Abstract

Nonlinear optical (NLO) switch materials have attracted considerable attention in photonics. Although various materials based on complex structural transitions have been developed extensively, the studies on light‐driven up‐conversion laser switches are rare, which have advantages including easy operations at room temperature and high contrasts. Here, the concept of photoswitch building unit is proposed to construct a novel sandwich‐like mixed‐matrix membrane. Dye@metal‐organic framework (MOF) crystals and spirooxazine are regarded as the laser emission and absorption units, followed by their hierarchical encapsulation into the polydimethylsiloxane carrier unit. Excited MOF microcrystals exhibit two‐photon pumped lasing anisotropy, with an ultrahigh degree of linear polarization (≈99.9%). Photochromic molecules can be interconverted by the external ultraviolet stimulus, causing sharp absorption‐band variations and inducing the laser emission or quenching. Such up‐conversion polarized laser switch material is reported for the first time. Record‐high NLO contrast (≈6.1 × 10^4^) among the solid‐state NLO switch materials can be obtained through simultaneously controlling the ultraviolet irradiation and the emission‐detected polarization direction at room temperature.

## Graphical abstract

A novel strategy toward designing light‐driven optical switch materials based on metal‐organic framework microlasers is developed through integrating various photoswitch building units into the sandwich‐like mixed‐matrix membranes. The resulting up‐conversion polarized laser switch is reported for the first time with the record‐high contrast among solid‐state nonlinear optical switch materials.

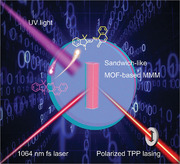

## 1 Introduction

Smart stimuli‐responsive optical materials, of which the physicochemical properties could be switched reversibly, have been extensively explored in scientific disciplines for decades.^[^ ^1^, ^2^, ^3^, ^4^ ^]^ Among them, those with controllable nonlinear optical (NLO) behaviors between on/off states are of great interest. NLO switch materials with high contrasts are fully expected in data storage, processing, and communications.^[^ ^5^, ^6^, ^7^ ^]^ Up to now, various solid‐state switch systems have been investigated, mostly in second‐harmonic generation switches, which generally undergo complicated structural transitions by heat treatment. These switching processes possibly suffer from serious service temperature constrains (normally above/below room temperature), undiversified control methods, and limited on/off contrasts.^[^ ^8^, ^9^, ^10^, ^11^ ^]^ By comparison, NLO materials with up‐conversion lasing performance might be much more advantageous, such as easy room temperature operations, high signal‐to‐noise ratio, and convenient‐to‐distinguish readout. Especially, the control of polarized lasers with enhanced emission contrasts is conducive to the analysis of the information brought by scattered light in radar remote sensing and the modulation in high‐efficiency displays or coherent optical communication.^[^ ^12^, ^13^, ^14^, ^15^ ^]^ However, such NLO switch materials are still rarely reported. It calls for deliberate materials selection and preparation strategy design.

In view of above considerations, up‐conversion polarized lasers switch materials with light as the stimulus are intended to be a reasonable solution. It is because light can precisely trigger a specific action with directional instruction, leading to the instant feedbacks and dramatic responses at room temperature.^[^ ^16^ ^]^ As a novel functional material, metal‐organic framework (MOF) is suggested to be an attractive light‐responsive candidate with designable structures.^[^ ^17^, ^18^, ^19^, ^20^ ^]^ On the one hand, the intermarriage of MOF and photochromic compounds creates an effective approach to realize spectral tuning.^[^ ^21^, ^22^, ^23^ ^]^ External light stimulus leads to the variation in the absorption spectra of photochromic molecules. Consequently, the overlap between the absorption band of photochromic molecules and MOF emission band directly determines the emission or quenching. On the other hand, MOF crystals with regular morphology and smooth surfaces could be used as natural laser resonant cavities. Their highly ordered porous structures impart strong spatial confinement to gain media, thereby enabling unique performances such as the anisotropic fluorescence emission or linearly polarized laser.^[^ ^24^, ^25^ ^]^ Nevertheless, the general methods to couple photochromic molecules with MOF are grinding and sublimation at high temperatures. They will bring irreversible destructions to the MOF resonant cavities.^[^ ^26^ ^]^

**Figure 1:**
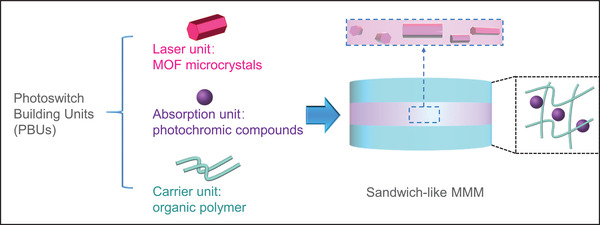
Conceptual representation of PBUs. MOF microcrystals, photochromic compounds, and polymer membrane function as three PBUs, corresponding to the laser emission, absorption, and carrier units. The photochromic molecules were mixed in the polymer. The sandwich‐like MMM was prepared by a layer‐by‐layer method and MOF microcrystals were only encapsulated in the second (middle) layer.

Therefore, we proposed the construction strategy of photoswitch building units (PBUs), as shown in **Figure** **1**. Multiple PBUs are integrated to prepare the sandwich‐like mixed‐matrix membrane (MMM). MOF microcrystals and photochromic compounds serve as the linearly polarized laser emission and the absorption units, respectively. Polymer is used as the carrier unit, where the two independent units mentioned above are encapsulated separately. This method ensures that the microlasers are surrounded by the photochromic molecules, without any possible extra destructions. Polarized laser can thus be switched to active or silent states via controlling the photoisomerization. Such MMM material not only integrates various individual PBUs intactly but also leads to enforced collaborative functions as expected.^[^ ^27^ ^]^

Herein, under the goal of realizing the upconversion polarized laser switches, we developed a new MOF‐based MMM by the introduction of PBUs. Organic dyes encapsulated MOF crystals, spirooxazine (SO), and polydimethylsiloxane (PDMS) are regarded as the laser emission, absorption, and carrier units, respectively. The aligned linear dye molecules are confined into the one‐dimensional (1D) MOF channels, resulting in the linearly polarized two‐photon pumped (TPP) lasing under the excitation of a 1064 nm femtosecond (fs) laser. The photochromic molecules are interconverted reversibly between two states, i.e., SO and merocyanine (MC) with sharp variations in red‐band absorption, by switchable external ultraviolet (UV) light exposure. TPP lasing could be quenched selectively by the MC state molecules. Ultrahigh NLO contrast (≈6.1 × 10^4^) is obtained by simultaneous control of UV irradiation on MMM and the emission‐detected polarization directions. Such novel composite material exhibits the record NLO contrast at room temperature in solid‐state NLO switch materials, which may open a simple and viable avenue for further development of high‐performance integrated optoelectronic devices.

## 2 Results and Discussion

### 2.1 Synthesis and Structure

**Figure 2:**
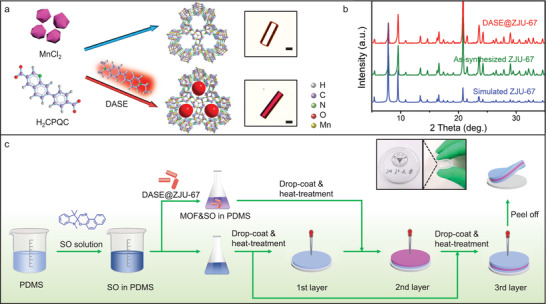
a) Schematic synthesis of ZJU‐67 and DASE@ZJU‐67 microcrystals. Insets: micrographs of the as‐synthesized ZJU‐67 and DASE@ZJU‐67 single crystals. Scale bars, 10 µm. b) Powder XRD patterns of the ZJU‐67 and DASE@ZJU‐67 materials. c) Schematic synthesis of the MOF‐based sandwich‐like MMM by a layer‐by‐layer stepwise method. Inset: the as‐obtained transparent MMM.

The solvothermal reaction of 7‐(4‐carboxyphenyl)quinoline‐3‐carboxylic acid (H_2_CPQC) and manganous chloride (MnCl_2_) in the mixed solvents led to the formation of a new MOF material, ZJU‐67. The colorless microcrystal possesses a regular hexagonal crystal morphology, as shown in **Figure** **2**a. Single crystal X‐ray diffraction studies reveal that ZJU‐67 crystallizes in P‐3 space group (Table S1, Supporting Information). Three Mn metal ions are assembled around a central µ_3_‐O atom to form trinuclear secondary building units (SBUs) of [Mn_3_O]^4+^ with the Mn–Mn distances of nearly 3.7 Å (Figure S1, Supporting Information). Each Mn atom is six‐coordinated and lies in an octahedral environment. These SBUs are linked by CPQC^2–^ ligands, forming an anionic framework of [Mn_3_O(C_17_H_9_NO_4_)_3_]^2–^ with 1D channels (the width of ≈6.5 Å) along the *c*‐axis (Figures S2 and S3, Supporting Information). ZJU‐67 exhibits the decomposition temperature above 450 °C (Figure S4, Supporting Information) and good chemical and thermal/photo‐induced structural stability (Figure S5, Supporting Information). A typical upconversion organic dye, DASE (E‐4(4‐dimethylaminostyryl)‐1‐ethylpyridinium) with the size of 6 × 14 Å^2^, was selected as the photonic guest and introduced into the host ZJU‐67. An in situ self‐assembly synthetic approach was employed and the as‐synthesized hybrid microcrystal represents a red appearance with the approximately 4.23 wt% dye content (Figure S6, Supporting Information). Figure 2b displays the powder XRD patterns of as‐synthesized ZJU‐67, DASE@ZJU‐67, and simulated ZJU‐67, respectively. The diffraction peaks of ZJU‐67 crystals with and without DASE encapsulation match well with the simulated one, which means the dye‐loading process could hardly destroy the ZJU‐67 crystal structure. According to the single crystal X‐ray diffraction result of DASE@ZJU‐67, linear DASE molecules are indeed aligned along the 1D channels due to the size matching and the strong confinement (Figure S7, Supporting Information).

MMM was prepared by integrating three individual PBUs, including DASE@ZJU‐67, SO, and PDMS. Such sandwich‐like material was created via a layer‐by‐layer stepwise preparation method (Figure 2c). A SO toluene solution was mixed in the liquid PDMS with a curing agent and the mixture was then divided into two parts, which were with and without the addition of DASE@ZJU‐67 microcrystals. Weighed mixture fluids were drop‐coated and heated in proper order for synthesizing the first, second, and third layers. The curing agent promoted the bridging reaction of PDMS molecules and solidification was completed by vacuum heat treatment. Finally, transparent three‐layer MMM could be peeled off from the Teflon substrate. Details can be found in the Experimental Section. Through observing the brittle fracture surfaces, the as‐prepared MMMs are uniform and dense, with the thickness of ≈2.0 mm (Figure S8, Supporting Information). Such MMM possesses flexible and foldable features (the insets of Figure 2c). DASE@ZJU‐67 crystals are only located in the second layer of such sandwich‐like MMM and coated completely by SO doped PDMS. Sufficient stirring will cause the materials to be uniformly dispersed in the MMM with the random and disorder arrangement (Figure S9, Supporting Information).

### 2.2 Optical Performances of PBUs

**Figure 3:**
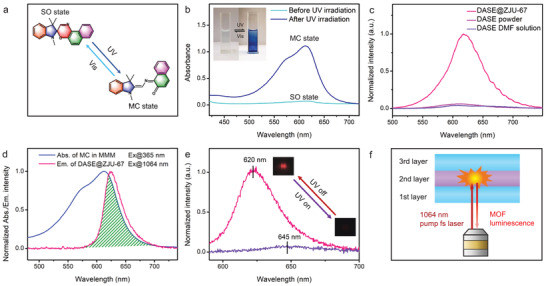
a) Reversible structural transformations of spirooxazines between SO and MC states by light stimuli. b) The absorption spectra of SO/MC doped PDMS, corresponding to the cyan and blue lines. The inset shows the obvious color change between transparent and deep blue states under the switchable UV light irradiation. c) The emission spectra of DASE dye and DASE@ZJU‐67 when excited at 480 nm. d) The absorption spectrum of MC doped membrane (blue line) and the emission spectrum of DASE@ZJU‐67 (red line), showing a large overlap (green area). e) The emission spectra of DASE@ZJU‐67 in MMM before and after external UV irradiation (correspond to red and purple lines, respectively) when the single crystal was excited by a 1064 nm fs laser. Insets: micrographs of the excited DASE@ZJU‐67 single crystal. Huge changes of emission intensity and wavelength are observed. f) Schematic diagram of the two‐photonluminescence switching process in the MMM. The 1064 nm fs laser passes through the first layer and excites DASE@ZJU‐67 crystals in the second layer. TPF is absorbed by the MC molecules in the sandwich‐like MMM and then collected.

As for the absorption unit, the well‐accepted switching process of SO photochromism is depicted in **Figure** **3**a. Briefly, upon UV light irradiation, the bond between the spiro carbon and the oxygen in the oxazine ring is rapidly broken to produce a ring‐opened isomer. The resulting structure is known as MC with characteristic absorption in the red‐band region due to an extended *π*‐system and the electron delocalization. It is worth emphasizing that when UV exposure is removed, MC will immediately revert to the initial SO in dark. Such fast optical switching process can be further accelerated by visible light (Vis) irradiation.^[^ ^28^, ^29^ ^]^ The photochromic behavior is clearly illustrated by comparing the UV‐vis absorption spectra at room temperature (Figure 3b). A new broad strong absorption peak centered at 612 nm indicates the successful conversion from SO to MC. Colorless SO doped PDMS changes to deep blue and recovers immediately, with and without a hand‐held UV lamp irradiating, respectively. The same phenomenon occurred in the as‐obtained transparent MMM (Figure S10, Supporting Information). No matter in room light or in dark, these fast reversible processes were all completed in short period of nearly 5 s (Figure S11, Supporting Information). The long‐wavelength band of MC can be recovered repeatedly during several cycles, indicating little loss of photochromic molecules over the course of these switching cycles (Figure S12, Supporting Information).

As for the emission unit, we compared the photoluminescent properties of MOF hybrid crystals with pure organic dyes. Both of DASE powder and solution (0.1 × 10^−3^ m) emit a faint fluorescence when excited at 480 nm (Figure 3c). In sharp contrast, DASE@ZJU‐67 hybrid microcrystals exhibit a significant emission enhancement. It is mainly attributed to the strong confinement, restricting intramolecular torsional motions and diminishing the aggregation‐caused quenching effects.^[^ ^17^ ^]^ The similar phenomenon could be observed under the excitation of a 1064 nm fs laser (Figure S13, Supporting Information). The fs laser beam is focused on the hexagonal MOF microcrystals in the MMM middle layer through the objective lens of a home‐built system (Figure S14, Supporting Information). A straight line with a slope of ≈2.03 (≈2.00) in the log‐log plots of integrated emission intensity versus pump energy density, confirms that such luminescence involved is a typical two‐photon fluorescence (TPF), due to the special donor‐(*π*‐spacer)‐acceptor structure of the linear photonic molecules DASE (Figure S15, Supporting Information). The calculated two‐photon action cross‐section of such MOF hybrid material is approximately 952 GM (details in Supporting Information).

Figure 3d reveals an obvious overlap between the TPF emission band and the MC absorption band, which may cause the TPF quenching. When the UV light irradiates the MMM, the original TPF intensity decreases rapidly and the central wavelength presents an obvious red shift from 620 to nearly 645 nm (Figure 3e). Once the UV lamp is turned off, it returns due to the fast transformation from MC to SO state. It proves that the red‐band TPF can be effectively absorbed by MC molecules, as elaborated in Figure 3f. Specifically, the pump fs laser passed through the first polymer layer and excited the DASE@ZJU‐67 microcrystals in the middle layer successfully. However, when the external UV light is employed, the TPF was absorbed by the surrounding MC molecules in the MMM, resulting in the as‐received extremely weak signal. Such energy flow process mainly relies on the photo reabsorption.^[^ ^30^ ^]^ In this process, SO doped PDMS could hardly absorb and affect the incident 1064 nm fs laser by comparing the as‐recorded laser energy under the switchable UV irradiation on the MMM (Figure S16, Supporting Information). We eliminated the influence of UV light itself to the excited MOF crystals through comparing TPF intensities before and after UV irradiation in a blank control MMM sample without adding any photochromic compounds. No obvious changes of TPF could be observed (Figure S17, Supporting Information).

### 2.3 Polarized TPP Lasing Study

**Figure 4:**
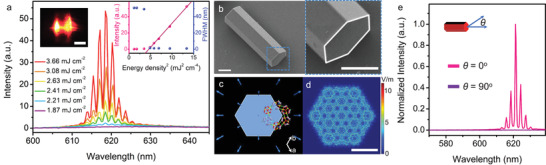
a) 1064 nm fs laser pumped emission spectra of DASE@ZJU‐67 with *R* = 6.32 µm under different pump energy densities. Insets: the micrograph of the excited DASE@ZJU‐67 single crystal (left); emission intensity and FWHM as functions of pump energy densities showing the lasing threshold of ≈1.95 mJ cm^–2^ (right). The scale bar, 10 µm. b) SEM images of the DASE@ZJU‐67 single crystal (left) and its hexagonal crystal section (right). Scale bars, 5 µm. c) Theoretically predicted morphology of the ZJU‐67 single crystal, showing the 1D channels along the *c*‐axis as well as the crystal longitudinal direction (the blue arrows direction), which is perpendicular to the crystal section. d) Simulated electric field distribution (*λ* = 618 nm, *n*_g_ = 2.96) in DASE@ZJU‐67 with *R* = 6.32 µm. The six crystal facets form the WGM‐type microcavity. The scale bar, 5 µm. e) Intensity‐dependent emission spectra from an excited DASE@ZJU‐67 microcrystals with emission‐detected polarization at two degree *θ* = 0^°^ and 90^°^ (the angle between the emission‐detected polarization direction and DASE@ZJU‐67 longitudinal direction was defined as *θ*, as shown in the inset), corresponding to the parallel and perpendicular directions to the crystal channels, respectively.

Enhanced luminescence properties are very conducive to the realization of optical gain and amplification. DASE molecules could serve as the photoactive gain media and ZJU‐67 microcrystals with smooth surfaces and morphology symmetry as the natural optical micro‐resonators. **Figure** **4**a summarizes the emission spectra under various energy densities and only TPF can be recorded at relatively low energy densities from the DASE@ZJU‐67 single crystal, optically pumped by a polarized 1064 nm fs pulse laser at room temperature. When the pump energy continues to increase, a set of sharp laser peaks appear immediately with a significant decrease of the full width at half maximum (FWHM) value from nearly 50.8 to 0.9 nm and a low threshold (*E*_th_) of ≈1.95 mJ cm^–2^ in the right inset. It presents strong emission with spatial interference from two‐side facets with two bright spots (the left inset), of which the patterns might belong to the whispering gallery modes (WGMs) feedback mechanism.^[^ ^31^ ^]^ In order to clarify the optical‐feedback mechanisms, a series of TPP lasing spectra from multiple selected DASE@ZJU‐67 microcrystals with different sizes were analyzed. The mode spacing (Δ*λ*) between adjacent lasing modes decreases with the increasing crystal side length *R*. As for WGMs laser, it is defined as

(1) $$\Delta \lambda =\lambda^{2}/n_{g}L$$

where *L*, *λ*, and *n*_g_ correspond to the cavity length as well as 3$\sqrt{3}$ *R*, the resonant wavelength, and the group refractive index, respectively.^[^ ^32^ ^]^ In this work, the measured Δ*λ* at 618 nm demonstrates good linear relationships with 1/*R*, which agrees well with the WGMs‐based equation (Figure S18, Supporting Information). The relatively high *n*_g_ value (≈2.96) is benefit for inducing strong self‐cavity optical confinement and optical feedback. Figure 4b presents the microscopy images of DASE@ZJU‐67 and the hexagonal cross‐section. It is consistent with the simulation results by using Bravais–Friedel–Donnay–Harker method, where the 1D channels direction (*c*‐axis) is parallel to the longitudinal direction of MOF crystal (Figure 4c). The six crystal facets form the WGMs‐type microcavity. The electric field distribution reveals that the energy of the photons could be confined well within the hybrid hexagonal cavity (Figure 4d). The internal reflection of six facets of DASE@ZJU‐67 can be observed clearly in the as‐simulated diagrams, which indicates the WGMs‐type resonances as well.

An obvious optical anisotropy was observed in the excited DASE@ZJU‐67 microcrystals. We tuned the polarization direction of the pump 1064 nm fs laser to the parallel position of the crystal longitudinal direction. The emitting light passed through a linear polarizer first, and was then collected. Here, the angle between the emission‐detected polarization direction and DASE@ZJU‐67 longitudinal direction (along *c*‐axis) was defined as *θ*. Figure 4e shows that when the detection direction is parallel to the crystal (*θ* = 0^°^), the emission intensity of TPP lasing reaches the maximum. However, TPP lasing converts to extremely weak TPF when it is perpendicular (*θ* = 90^°^). Such switchable TPP lasing signals could last several cycles (Figure S19, Supporting Information). Degree of polarization can be defined as DOP by the equation:^[^ ^33^ ^]^

(2) $$\mathrm{DOP}=\left(I_{//}-I_{⊥}\right)/\left(I_{//}+I_{⊥}\right)$$

where *I*_//_ and *I*_⊥_ are the corresponding emission intensities monitored at orthogonal *θ*. The DOP value comes to ≈99.9% in this upconversion microlaser. The polarization characteristics of the microcavity laser are mainly determined by two aspects. On the one hand, importantly, the host ZJU‐67 with 1D channels efficiently confine guest single‐dipole DSAE molecules (the absorption transition moments approximately along the linear dye molecules axis) to achieve highly oriented alignment of absorption/emission dipoles, which defines from the source the emission polarization direction. It could be further confirmed by the polar plot, which reveals an obvious contrast among the emission intensities under different excitation polarization directions (Figure S20, Supporting Information). On the other hand, the hexagonal prism‐like resonator could provide polarization selection mechanism for WGMs feedback emission, resulting in the final anisotropic lasing output.^[^ ^34^, ^35^, ^36^, ^37^ ^]^

### 2.4 Polarized TPP Lasing Switch

**Figure 5:**
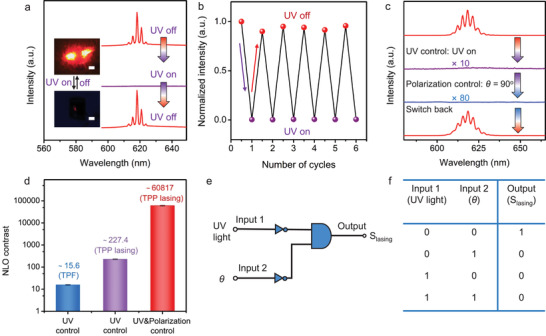
a) 1064 nm pumped TPP lasing spectra from the excited single DASE@ZJU‐67 crystal in the MMM with and without UV irradiation (when *θ* = 0^°^). Insets: micrographs of the excited DASE@ZJU‐67 single crystal. Scale bars, 10 µm. b) Visualization of the switching cycles. The emission intensity of excited DASE@ZJU‐67 alters greatly when the states (on/off) of the external UV light are changed. c) Switchable TPP lasing spectra from the excited single DASE@ZJU‐67 crystal in the MMM by simultaneous control of UV light and the emission‐detected polarization direction. When the external UV light is on and *θ* is tuned to 90^°^, the TPP lasing disappears and almost no signals can be recorded. It recovers when these controls are cancelled. (UV control: UV light irradiation on/off control; polarization control: emission‐detected polarization direction control) d) NLO contrasts of TPF and TPP lasing from the excited DASE@ZJU‐67 single crystal under different control conditions. The values are 15.6 (±5.3%), 227.4 (±4.5%), and 60 817 (±5.3%), respectively. e) The logic gate represented by a conventional gate natation. The external UV light and emission‐detected polarization direction (*θ*) are regarded as the input 1 and 2, and the TPP lasing signal as the output. f) The truth table for such logic gate.

There is a significant overlap between lasing emission band and the absorption band of the MC molecules (Figure S21, Supporting Information), which provides an opportunity to induce or quench the TPP lasing emission by the switchable external UV light irradiating on the MMM (Figure S22, Supporting Information). Through controlling the external UV stimulus from a hand‐held 365 nm lamp, polarized TPP lasing is switched between active and silent states with sharp spectral variations (in the situation of *θ* = 0^°^), as shown in **Figure** **5**a. To be specific, when the UV lamp is on, the sharp peaks suddenly disappear, while they revert when the lamp is off. The excited microcrystal in MMM exhibits great contrasts in the micrographs of the inset. Figure 5b illustrates the switching cycles between extremely weak TPF and intense TPP lasing signals for the further feasibility verification of such MMM‐based optical switch. Through analyzing the continuous spectral information recorded by the fiber optic spectrometer, the emission intensity displays a close dependence on the UV light stimulus, illustrating the average responsive times of ≈1.1 and 1.9 s for turning off/on, respectively. This phenomenon could be observed not only in room light but also in dark (Figures S23 and S24, Supporting Information). To our knowledge, this result of fast response is one of the best in those of MOF materials reported up to now (Table S2, Supporting Information). In this case, if *θ* is further tuned to 90^°^, the as‐received TPF signal disappears (limited by the spectral intensity sensitivity), as shown in Figure 5c. It is attributed to the optically anisotropic emission of the DASE@ZJU‐67 as mentioned above. The TPP lasing signal could be switched back when both UV stimulus and polarization control were cancelled. The interference of UV light to the TPP lasing can be eliminated by testing the blank control MMM sample (Figure S25, Supporting Information).

NLO contrast is defined by the on/off ratio in order to clarify and evaluate the function of NLO switch materials.^[^ ^38^ ^]^ In the excited DASE@ZJU‐67 single microcrystal, NLO contrast of TPF under the UV control is ≈15.6. However, the value for TPP lasing is ≈227.4, as shown in Figure 5d. It is mainly attributed to the up‐conversion laser with the advantages of more intense emission strength and higher signal‐to‐noise ratio. With the simultaneous control of UV light and the emission‐detected polarization direction, the contrast comes to ≈60 817 (limited by the instrument sensitivity of fiber spectrometer). To our knowledge, it is the record value among the reported solid‐state NLO switch materials (Table S3, Supporting Information). In summary, such MOF‐based NLO switch material possesses significant advantages. Firstly, switchable TPP lasing with sharp narrow emission peaks and extremely intense laser could lead to the easy‐to‐recognize signals and high contrasts. Secondly, as a convenient, useful, and harmless stimulus, light can trigger a specific action rapidly at room temperature, preventing the NLO switching process from complex structural transformations. Thirdly, the cooperation of linearly polarized micro‐laser with photochromic compounds makes such optical switch using the sandwich‐like MMMs enable to be manipulated by dual control of external UV light and the emission‐detected polarization direction, thereby enriching the control methods and increasing the NLO contrasts. Finally, the flexible structure endows such MMMs with tailorable characteristics and the membranes could be engineered with any sizes on demand (Figure S26, Supporting Information). This novel preparation method is simple and practical, which can provide reference to the application of NLO materials in optical switches.

Furthermore, such NLO switch materials exhibit great potentials in the field of logic gates. The complete pictorial representation and truth table are given in Figure 5e,f. Logic gates could be established by using two input signals of the external UV light (input 1) and the emission‐detected polarization direction (input 2), respectively. As for input 1, the off/on states of UV light could be regarded as binary “0” and “1”. As for input 2, the situations of *θ* = 0^°^ and 90^°^ are defined as “0” and “1”, respectively. We regarded the absence and presence of TPP lasing as the outputs, which can be encoded as “0” and “1”. Only if (input1, input2) is (0, 0), TPP lasing signals could be recorded, corresponding to output signal “1”. Otherwise, the output signals are “0” under the other input conditions. This simple method provides an opportunity to design novel programmable logic devices based on NLO switches at room temperature.

## 3 Conclusions

In conclusion, we developed a novel up‐conversion polarized laser switch based on photoresponsive MMMs with the integration of multiple PBUs. DASE@ZJU‐67 microlasers unit achieved TPP lasing with a high degree of linear polarization ≈99.9% and the intensity can be manipulated by tuning the emission‐detected polarization directions. Sharp variations in red‐band absorption between MC and SO in MMM are caused by switchable UV irradiation. TPP lasing can be absorbed and quenched selectively by the MC state molecules, not the SO ones. Upconversion polarized laser switches are therefore realized by precise manipulation of the external UV light and emission‐detected polarization at room temperature, resulting in the record‐high NLO contrast (≈6.1×10^4^) among the solid‐state NLO switch materials. We believed that such MOF‐based sandwich‐like MMMs with unique polarized upconversion laser switching properties could provide a new strategy and powerful platform to develop smart photonic devices with outstanding performances in the future.

## 4 Experimental Section

### Fabrication of ZJU‐67

MnCl_2_·4 H_2_O (20 mg) and H_2_CPQC (5 mg) were dissolved in the solution of *N*,*N*‐dimethylformamide (DMF, 1 mL), acetonitrile (0.25 mL), and H_2_O (0.05 mL). The mixture was transferred into a 20 mL glass vial and then heated at 100 ^ °^C for 24 h. The as‐prepared crystals were collected by filtration, then washed with DMF and ethanol (EtOH), and then dried in air.

### Fabrication of DASE@ZJU‐67

The mixture mentioned above with DASE (1 mg) was heated at 100  °C for 24 h and then rapidly cooled to room temperature. The hybrid MOF crystals were collected by filtration and rinsed several times with fresh DMF and EtOH in order to completely remove the residual dye on the surfaces.

### Fabrication of the MMM

A mixture of liquid PDMS (Sylgard DC184) and curing agent (weight ratio 10: 1) is stirred for 30 min by agitating magnetically at room temperature. Then a toluene solution of SO (25 mg in 0.5 mL) was added, stirring for more 30 min. The mixture was divided into two parts. An amount of 1.0 g of the first part was dropped onto the pre‐cleaned Teflon substrate (the diameter ≈4 cm). The sample was placed on a horizontal table, standing for 10 min. Then, it was put into the vacuum oven and the solidification was completed in 1 h at 80 °C after vacuum degassing process. DASE@ZJU‐67 crystals (10 mg dispersed in 0.5 mL toluene) were added into the second part mixture. After stirring for 30 min, 1.0 g of this mixed fluid was partly dropped onto the first layer. The above treatment steps were repeated until the solidification was finished. Finally, another 1.0 g of the first part mixture was dropped onto the second layer for further treatment. After total solidification, three‐layer sandwich‐like MMM could be peeled off successfully from the Teflon substrate. The process diagram is presented in Figure S27 (Supporting Information). The appropriate addition of MOF can ensure the uniform distribution of microcrystals in MMM. An amount of 10 mg MOF (hundreds of microcrystals) is more than enough for the tests. For the TPP lasing and optical switching tests, the MMMs were tailored and divided into multiple rectangular membranes with the sizes of approximately 5 × 5 mm^2^ (Figure S28, Supporting Information).

### Fabrication of the Blank Control Sample MMM

The same method to prepare the MOF‐based MMM mentioned above was used. No photochromic compound was added.

### Light Sources Used in the Test

As for the pump light, an optical parametric amplifier (Spirit‐OPA + Spirit‐OPA‐UV3, Newport Corporation) was pumped by a fully automated ultrafast laser system (Spirit One 1040–8, 8W at 1040 nm, Newport Corporation), which was used for generating the excitation pulse (1 kHz, 1064 nm, pulse width < 400 fs). The on/off states of external UV light were controlled by a hand‐held 365 nm UV lamp (300 mW). The emission band of indoor light (>420 nm) was measured (Figure S29, Supporting Information).

[CCDC 2 114 954 (ZJU‐67) contains the supplementary crystallographic dates for this paper. These data can be obtained free of charge from The Cambridge Crystallographic Data Centre via www.ccdc.cam.ac.uk/data_request/cif.]

## Conflict of Interest

The authors declare no conflict of interest.

## Supporting information

Supporting Information

## Data Availability

The data that support the findings of this study are available in the supplementary material of this article.

## References

[1] A. J. McConnell , C. S. Wood , P. P. Neelakandan , J. R. Nitschke , Chem. Rev. 2015, 115, 7729. 25880789 10.1021/cr500632f

[2] P. Hosseini , C. D. Wright , H. Bhaskaran , Nature 2014, 511, 206. 25008527 10.1038/nature13487

[3] A. F. Koenderink , A. Alu , A. Polman , Science 2015, 348, 516. 25931548 10.1126/science.1261243

[4] A. A. Zhumekenov , M. I. Saidaminov , O. F. Mohammed , O. M. Bakr , Joule 2021, 5, 2027.

[5] J. Clark , G. Lanzani , Nat. Photonics 2010, 4, 438.

[6] Z. Sun , D. W. Snoke , Nat. Photonics 2019, 13, 370.

[7] W. J. Xu , C. T. He , C. M. Ji , S. L. Chen , R. K. Huang , R. B. Lin , W. Xue , J. H. Luo , W. X. Zhang , X. M. Chen , Adv. Mater. 2016, 28, 5886. 27159779 10.1002/adma.201600895

[8] S. Y. Zhang , X. Shu , Y. Zeng , Q. Y. Liu , Z. Y. Du , C. T. He , W. X. Zhang , X. M. Chen , Nat. Commun. 2020, 11, 2752. 32487992 10.1038/s41467-020-15518-z PMC7265397

[9] Y. Zeng , C. L. Hu , W. J. Xu , T. W. Zeng , Z. X. Zhu , X. X. Chen , D. X. Liu , Y. J. Chen , Y. B. Zhang , W. X. Zhang , X. M. Chen , Angew. Chem., Int. Ed. 2022, *61*, e202110082. 10.1002/anie.202110082 34653302

[10] C. Y. Pan , X. R. Yang , L. Xiong , Z. W. Lu , B. Y. Zhen , X. Sui , X. B. Deng , L. Chen , L. M. Wu , J. Am. Chem. Soc. 2020, 142, 6423. 32160462 10.1021/jacs.0c01741

[11] W.‐F. Chen , B.‐W. Liu , S.‐M. Pei , Q.‐N. Yan , X.‐M. Jiang , G.‐C. Guo , Chem. Mater. 2021, 33, 3729.

[12] Z. Gao , K. Wang , Y. Yan , J. Yao , Y. S. Zhao , Natl. Sci. Rev. 2021, 8, nwaa162. 34691572 10.1093/nsr/nwaa162 PMC8288339

[13] C. Huang , C. Zhang , S. Xiao , Y. Wang , Y. Fan , Y. Liu , N. Zhang , G. Qu , H. Ji , J. Han , L. Ge , Y. Kivshar , Q. Song , Science 2020, 367, 1018. 32108108 10.1126/science.aba4597

[14] J. Hu , L. Li , W. Yang , L. Manna , L. Wang , A. P. Alivisatos , Science 2001, 292, 2060. 11337589 10.1126/science.1060810

[15] E. Matioli , S. Brinkley , K. M. Kelchner , Y.‐L. Hu , S. Nakamura , S. DenBaars , J. Speck , C. Weisbuch , Light: Sci. Appl. 2012, 1, e22.

[16] Z. Chai , X. Hu , F. Wang , X. Niu , J. Xie , Q. Gong , Adv. Opt. Mater. 2017, 5, 1600665.

[17] R. Medishetty , J. K. Zareba , D. Mayer , M. Samoc , R. A. Fischer , Chem. Soc. Rev. 2017, 46, 4976. 28621347 10.1039/c7cs00162b

[18] Z. Chen , P. Li , R. Anderson , X. Wang , X. Zhang , L. Robison , L. R. Redfern , S. Moribe , T. Islamoglu , D. A. Gomez‐Gualdron , T. Yildirim , J. F. Stoddart , O. K. Farha , Science 2020, 368, 297. 32299950 10.1126/science.aaz8881

[19] N. Hanikel , X. Pei , S. Chheda , H. Lyu , W. Jeong , J. Sauer , L. Gagliardi , O. M. Yaghi , Science 2021, 374, 454. 34672755 10.1126/science.abj0890

[20] E. J. Kim , R. L. Siegelman , H. Z. H. Jiang , A. C. Forse , J.‐H. Lee , J. D. Martell , P. J. Milner , J. M. Falkowski , J. B. Neaton , J. A. Reimer , S. C. Weston , J. R. Long , Science 2020, 369, 392. 32703872 10.1126/science.abb3976 PMC8262103

[21] F. Bigdeli , C. T. Lollar , A. Morsali , H. C. Zhou , Angew. Chem., Int. Ed. 2020, 59, 4652. 10.1002/anie.201900666 31134738

[22] R. Haldar , L. Heinke , C. Woll , Adv. Mater. 2020, 32, 1905227. 10.1002/adma.201905227 31763731

[23] A. M. Rice , C. R. Martin , V. A. Galitskiy , A. A. Berseneva , G. A. Leith , N. B. Shustova , Chem. Rev. 2019, 120, 8790. 31638383 10.1021/acs.chemrev.9b00350

[24] H. Li , L. Zhang , H. He , E. Hu , Y. Yang , B. Li , Y. Cui , G. Qian , Adv. Opt. Mater. 2020, 8, 2001089.

[25] D. Kottilil , M. Gupta , C. Vijayan , P. K. Bharadwaj , W. Ji , Adv. Funct. Mater. 2020, 30, 2003294.

[26] N. Kulachenkov , Q. Haar , S. Shipilovskikh , A. Yankin , J. F. Pierson , A. Nominé , V. A. Milichko , Adv. Funct. Mater. 2021, 32, 2107949.

[27] T. Kitao , Y. Zhang , S. Kitagawa , B. Wang , T. Uemura , Chem. Soc. Rev. 2017, 46, 3108. 28368064 10.1039/c7cs00041c

[28] T. Ubukata , S. Fujii , K. Arimatsu , Y. Yokoyama , J. Mater. Chem. 2012, 22, 14410.

[29] D. Iacopino , G. Redmond , Chem. Commun. 2011, 47, 9170. 10.1039/c1cc12930a 21755084

[30] G. Zhu , J. Li , P. Li , Z. Tian , J. Dai , Y. Wang , Z. Shi , C. Xu , Europhys. Lett. 2015, 110, 67007.

[31] H. He , E. Ma , Y. Cui , J. Yu , Y. Yang , T. Song , C. D. Wu , X. Chen , B. Chen , G. Qian , Nat. Commun. 2016, 7, 11087. 26983592 10.1038/ncomms11087 PMC4800435

[32] Y. Wei , H. Dong , C. Wei , W. Zhang , Y. Yan , Y. S. Zhao , Adv. Mater. 2016, 28, 7424. 27314453 10.1002/adma.201601844

[33] J. C. Johnson , H. Q. Yan , P. D. Yang , R. J. Saykally , J. Phys. Chem. B 2003, 107, 8816.

[34] Y. Liang , H. Zhu , H. Zheng , Z. Tang , Y. Wang , H. Wei , R. Hong , X. Gui , Y. Shen , J. Phys. D: Appl. Phys. 2021, 54, 055107.

[35] J. Lu , F. Li , W. Ma , J. Hu , Y. Peng , Z. Yang , Q. Chen , C. Xu , C. Pan , Z. L. Wang , Adv. Sci. 2019, 6, 1900916. 10.1002/advs.201900916 PMC6864518 31763135

[36] J. Dai , C. X. Xu , K. Zheng , C. G. Lv , Y. P. Cui , Appl. Phys. Lett. 2009, 95, 241110.

[37] C. Xu , J. Dai , G. Zhu , G. Zhu , Y. Lin , J. Li , Z. Shi , Laser Photonics Rev. 2014, 8, 469.

[38] Z. Sun , J. Luo , S. Zhang , C. Ji , L. Zhou , S. Li , F. Deng , M. Hong , Adv. Mater. 2013, 25, 4159. 23813746 10.1002/adma.201301685

