# Supplementary material for: Polarized Laser Switching with Giant Contrast in MOF‐Based Mixed‐Matrix Membrane
Source: Adv Sci (Weinh). 2022 Apr 11;9(17):2200953. doi: 10.1002/advs.202200953 (PMC9189632; doi:10.1002/advs.202200953)
Supplement: Supplementary file 1 — Supporting Information [file ADVS-9-2200953-s001.pdf]

ADVANCED SCIENCE
Open Access

Supporting Information

for *Adv. Sci.*, DOI 10.1002/advs.202200953

Polarized Laser Switching with Giant Contrast in MOF-Based Mixed-Matrix Membrane

*Hongjun Li, Lin Zhang, Yu Yang, Enlai Hu, Bin Li, Yuanjing Cui, Deren Yang and Guodong Qian**

WILEY-VCH

Copyright Wiley-VCH GmbH

Supporting Information

Polarized Laser Switching with Giant Contrast in MOF-based Mixed-matrix Membrane

*Hongjun Li, Lin Zhang, Yu Yang, Enlai Hu, Bin Li, Yuanjing Cui, Deren Yang, and Guodong Qian**

E-mail: gdqian@zju.edu.cn

Contents

1. Experimental Section
2. Supporting Figures and Tables

1. Experimental Section

Material Characterizations: Powder X-Ray diffraction (XRD) data were measured on an X'Pert PRO diffractometer (Cu Kα radiation (λ = 1.542 Å), room temperature, in the range of 2θ = 3 to 50°). Thermogravimetric analyses (TGAs) were taken on a Netzch TG209F3 with heating rate of 5 °C min^{-1} in N_2 atmosphere. UV-Vis spectroscopy was recorded on Shimadzu UV-2600. Photoluminescence spectra were collected with a Hitachi F-4600 fluorescence spectrometer. The MMM morphology was elucidated by FESEM carried out on a Hitachi S-4800 field emission scanning electron microscope. For lasing experiments, the incident polarized 1064 nm fs laser was coupled to the microscope (IX71, Olympus) and focused on crystals through an objective lens (Olympus LUCPlanFL N 10×, numerical aperture = 0.40). The emitting light was focused and collected by the fiber optic spectrometer (PG2000-Pro, Ideaoptics Instruments). The continuous spectral information was also obtained in such spectrometer by the mode of Time Sequence with the minimum time interval of 100 ms. Lasing emission anisotropy test experiment was carried on when the polarization direction of 1064 nm pump fs laser was parallel to the DASE@ZJU-67 longitudinal direction (along *c*-axis). Through rotating a linear polarizer in the receiving light path, the emission-detected polarization detection was changed and the emission intensity at orthogonal θ could be collected by the fiber optic spectrometer. The propose of this test is to verify whether the emitting light is anisotropic. The polar plot was obtained through simply changing the polarization direction of the pump laser. A linear polarizer was kept on the path in order to ensure a constant input 1064 nm laser polarization. An appropriate half-wave plate was used to rotate the incident polarization. The propose of this test is to verify the polarization direction of the sample. Both of these two tests are useful for examining the optical anisotropy and widely employed in this field.[1-4] The simulation of crystal channel and morphology is completed by software Materials Studio. The electric field distribution is completed by using software COMSOL Multiphysics (version 5.6).

Single crystal testing: Single crystal data of **ZJU-67** was obtained by a Bruker APEX-II diffractometer with a CCD detector and phi and omega scans using graphite-monochromated Mo-K radiation (λ = 0.71073 Å) at 293K.

Two-photon action cross section testing: A typical method, which is widely accepted, is used.[5-8] The formula below was used to estimate the two-photon action cross section of DASE@ZJU-67. Rhodamine 6G (R6G) ethanol solution (10^{-4} M) was used as the reference,

$$\eta_{(s)}\sigma_{(s)} = (F_{(s)}/F_{(r)})(\rho_{(r)}/\rho_{(s)})\eta_{(r)}\sigma_{(r)}$$

where $F_{(s)}$ and $F_{(r)}$ are the measured DASE@ZJU-67 microcrystals sample and the two-photon fluorescence signal of R6G solution reference, respectively. For such two signals, the pump laser wavelength is selected as 1060 nm (20 kHz) and all the test conditions are the same. η is the quantum yield, σ is the two-photon absorption cross section, and $\eta\sigma$ is the two-photon action cross section. ρ is the molar concentration. The $\sigma_{(reference)}$ of the known two-photon standard, R6G is reported to be 9.9 GM (9.9×10^{-50} $cm^4\cdot s\cdot photon^{-1}$) at 1060 nm and the $\eta_{(r)}$ of R6G 10^{-4} M ethanol solution is ~ 89.5%. These results could be found in the related studies.[9-11] The molar concentration of DASE in ZJU-67 could be calculated by the formula:

$$\rho_{(s)} = 2R/N_A V$$

where R could be obtained from the formula of DASE@ZJU-67, which is $(DASE)_R H_{2-R}[Mn_3O(C_{17}H_9NO_4)_3]$. According to the as-obtained dye content (mass fraction, 4.23 wt%), the value of R is approximately 0.177. In ZJU-67, V = 2481.2 $Å^3$ and $N_A = 6.02 \times 10^{23}$ mol^{-1}. After calculation, $\rho_{(s)}$ = 0.237 mM. Therefore, the value of $\eta\sigma_{(s)}$ is approximately 952 GM, which is larger than those of dye solution.[12-14]

Table S1.

Crystallographic Data for ZJU-67.

Compound	**ZJU-67**
Empirical formula	$C_{51}H_{29}Mn_3N_3O_{13}$
CCDC	2114954
Formula weight	1056.59
Temperature (K)	268(2)
Crystal system	Trigonal
Space group	P-3
a (Å)	13.1776(16)
b (Å)	13.1776(16)
c (Å)	16.499(2)
α (°)	90.00
β (°)	90.00
γ (°)	120.00
Volume (Å^3)	2481.2(7)
Z	2
$\rho_{(calc)}$ (g/cm^3)	1.414
μ (mm^{-1})	0.818
F (000)	1070.0
Radiation	MoKα (λ = 0.71073)
2θ range for data collection (°)	6.094 to 56.714
Index ranges	$-17 \leq h \leq 17$, $-17 \leq k \leq 17$, $-22 \leq l \leq 19$
Reflections collected	55119
Independent reflections	4133 [R_{int} = 0.0577, R_{sigma} = 0.0240]
Data/restraints/parameters	4133/0/212
Goodness-of-fit on F^2	1.071
Final R indexes [I>=2σ (I)]	R_1 = 0.0374, wR_2 = 0.0981
Final R indexes [all data]	R_1 = 0.0423, wR_2 = 0.1012
Largest diff. peak/hole (e Å^{-3})	0.73/-0.66

Table S2.

Comparison of the NLO contrasts and the response times in the reported MOF-based photoswitch materials.

	MOF	On/off Contrast	Material states	Time (s)	Ref.
1	ZJU-88	10	single crystal	60–90	[15]
2	ZIF-8	10	single crystal	1800	[16]
3	HKUST-1	≈ 5	single crystal	0.005	[17]
4	$Zn_2(ZnTCPP)$	≈ 1.7	single crystal	1800–6420	[18]
5	HKUST-1	1.16	thin film	300	[19]
6	ZIF-70	1.1	single crystal	600	[20]
7	ZnMOF-74	–	thin film	10	[21]
8	Ni-NDISA	–	thin film	7	[22]
9	HKUST-1	–	thin film	5	[23]
10	CuTCA	–	thin film	5	[23]
11	$Cu_3(HHTP)_2$	–	thin film	3	[24]
12	ZJU-67	≈ 227.4	single crystals in MMM	≈ 1.1–1.9	This work

Table S3.

Comparison of the NLO contrasts and the switching methods in the reported solid-state NLO switch materials. SHG: second-harmonic generation.

	Material	Method	NLO response	NLO Contrast	Ref.
1	KSb_5S_8, $RbSb_5S_8$	Crystal-glass transformation	SHG	365.6, 664.5	[25]
2	Imidazolium-$FCrO_3$	Phase transitions with T_c (240 K)	SHG	≈ 250	[26]
3	2-(hydroxymethyl)-2-nitro-1,3-propanediol	Phase transitions with T_c (340 K)	SHG	≈ 150	[27]
4	(i-$PrNHMe_2$)[Cd$(SCN)_3$]	Phase transitions with T_s (273–328 K)	SHG	≈ 74	[28]
5	$\{[Pb_3Cl_6(CV)]\cdot H_2O]\}_n$	hν/60°C, O_2	SHG	≈ 67	[29]
6	$[(EPCF)_x(EPCCl)_{1-x}][Mn(dca)_3]$	Phase transitions with T_s (268–282 K)	SHG	≈ 46	[30]
7	$[C_5H_{12}N]SnCl_3$	Phase transitions with T_c (383 K)	SHG	≈ 40	[31]
8	$[Et_3(n\text{-}Pr)P][Cd(dca)_3]$	Phase transitions with T_2 (386 K)	SHG	≈ 40	[32]
9	MOF NH_2-MIL-53(Al)	Gas treatment	SHG	≈ 40	[33]
10	$(C_4H_{16}N_3)BiBr_6$	Phase transitions with T (335 K)	SHG	≈ 35	[34]
11	$(Hdabco^+)(CF_3COO^-)$	Phase transitions with T_c (181 K)	SHG	≈ 35	[35]
12	$(Me_3NNH_2)_2[CdI_4]$	Phase transitions with T (213–303K)	SHG	≈ 25.5/4.3/9.2	[36]
13	$[H_2dabcoCl_2][FeCl_3(H_2O)_3]$	Phase transitions with T_c (279.9 K)	SHG	≈ 25	[37]
14	$\{[(CBbpy)Zn_3(TBC)(HTBC)(OH)]\cdot NO_3\}n$	UV irradiation and heating treatments	SHG	≈ 23	[38]
15	N-methylcyclohexylaminium picrate	Phase transitions with T_1 (240K)	SHG	≈ 20	[39]
16	Surface-stabilized ferroelectric liquid-crystalline polymers	Two-photon isomerization of azobenzene molecules.	SHG	≈ 20	[40]
17	$(C_6H_{14}N)_2SbCl_5$	Phase transitions with T_c (335 K)	SHG	≈ 13	[41]
18	bis-(imidazolium hydrochlorate) dihydrate 18-crown-6	Phase transitions with T_c (219K)	SHG	≈ 12	[42]
19	$[(DPA)(18\text{-crown-}6)]ClO_4$	Phase transitions with T_c (214K)	SHG	≈ 10	[43]
20	$K_x(NH_4)_{2-x}PO_3F$	Phase transitions with T_c (270-150K)	SHG	> 8	[44]
21	Dithienylethene-based platinum(II) complexes	UV/Vis irradiation	SHG	≈ 5	[45]
22	Photochromic zinc(II) compound	hν/dark	SHG	≈ 3.5	[46]
23	MOF-based MMM	Switchable UV control at room temperature	TPP lasing	≈ 227.4	This work
		Switchable UV &polarization control at room temperature	TPP lasing	≈ 60817	This work

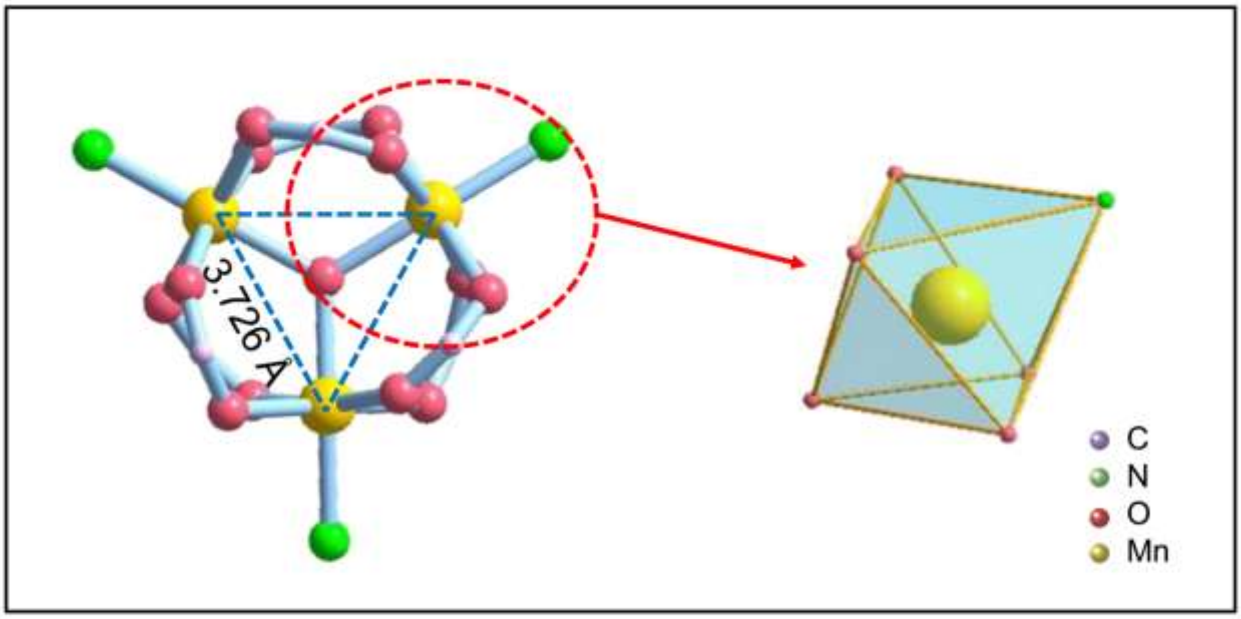

Figure S1.
SBUs of ZJU-67 (left) and the coordination environment of Mn atom (right). Each Mn atom is six-coordinated and lies in an octahedral environment.

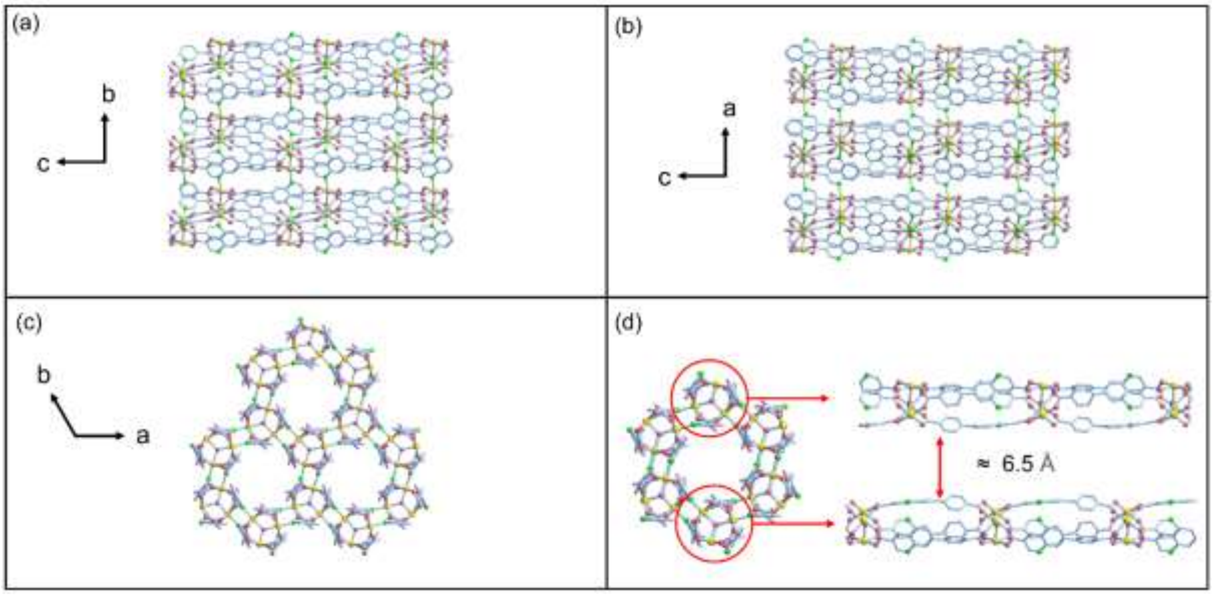

Figure S2.
(a-c) X-Ray crystal structure of ZJU-67 as viewed along *a*-axis (a), *b*-axis (b) and *c*-axis (c). (d) 1-D channel along *c*-axis with the width of approximately 6.5 Å.

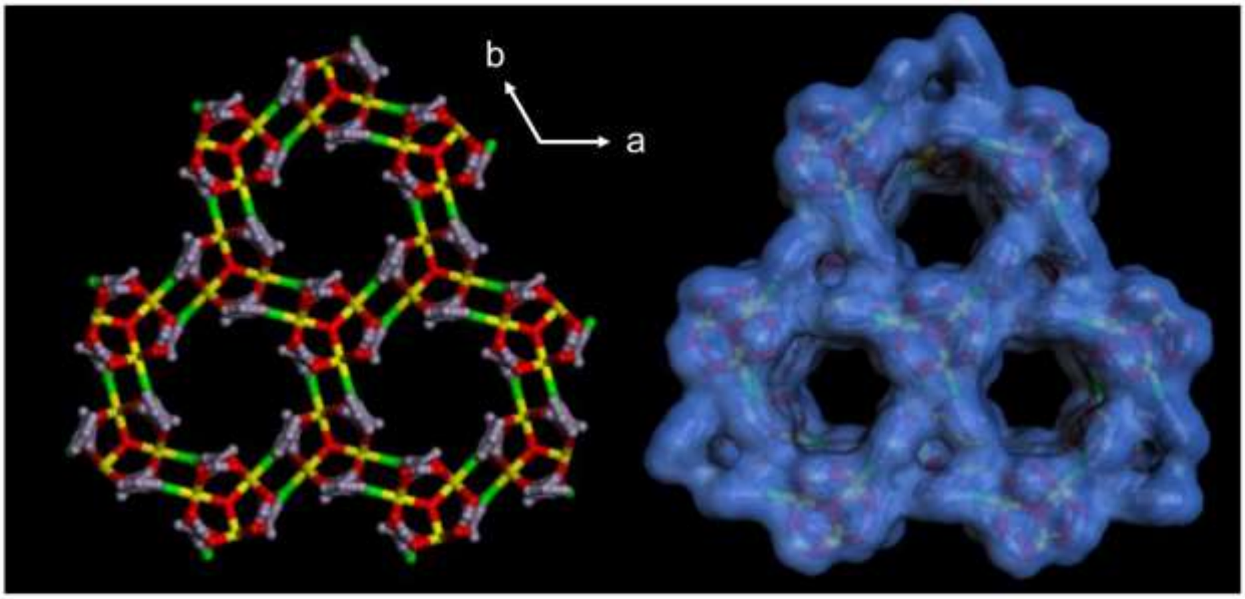

Figure S3.

Simulation of pore structures of the ZJU-67 crystal by Materials Studio. It displays the 1-D channels along *c*-axis.

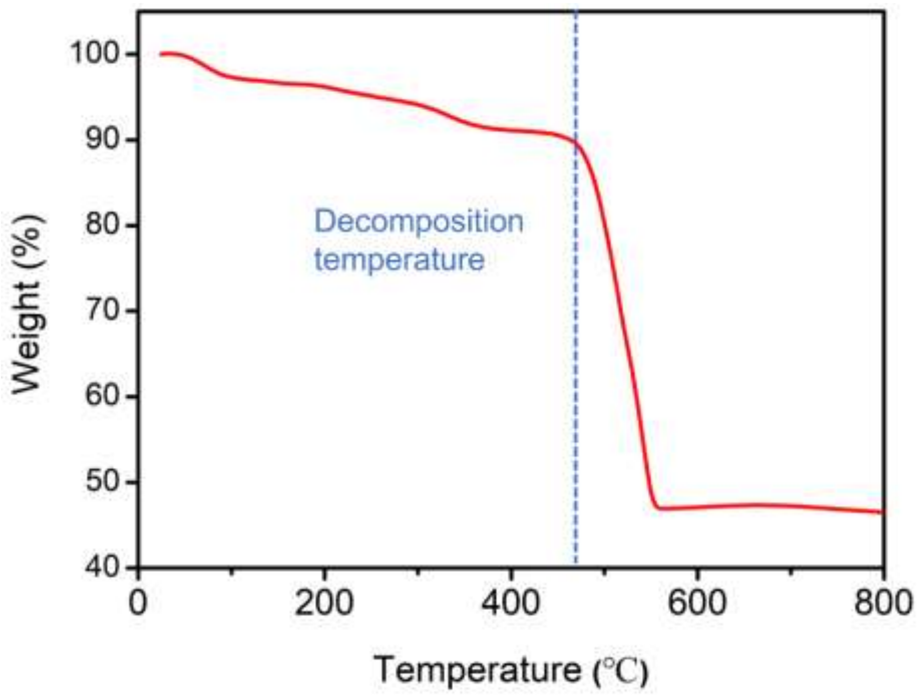

Figure S4.
TGA curve of as-synthesized ZJU-67.

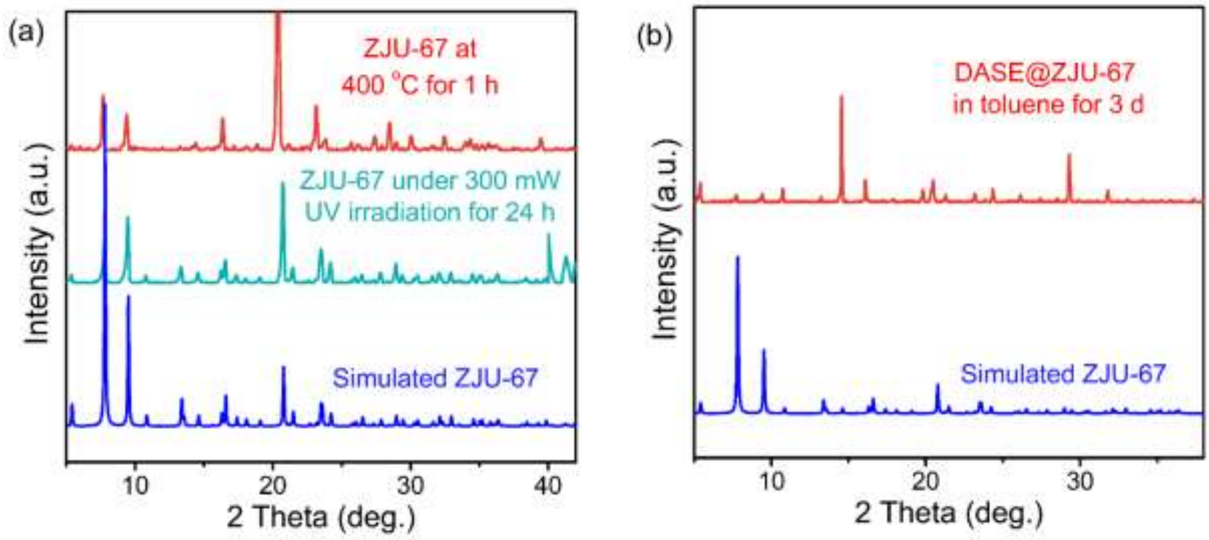

Figure S5.

(a). PXRD patterns of ZJU-67 after UV irradiation and heat treatment at 400 ^{o}C. (b). PXRD patterns of DASE@ZJU-67 after keeping in toluene solvent for 3 days.

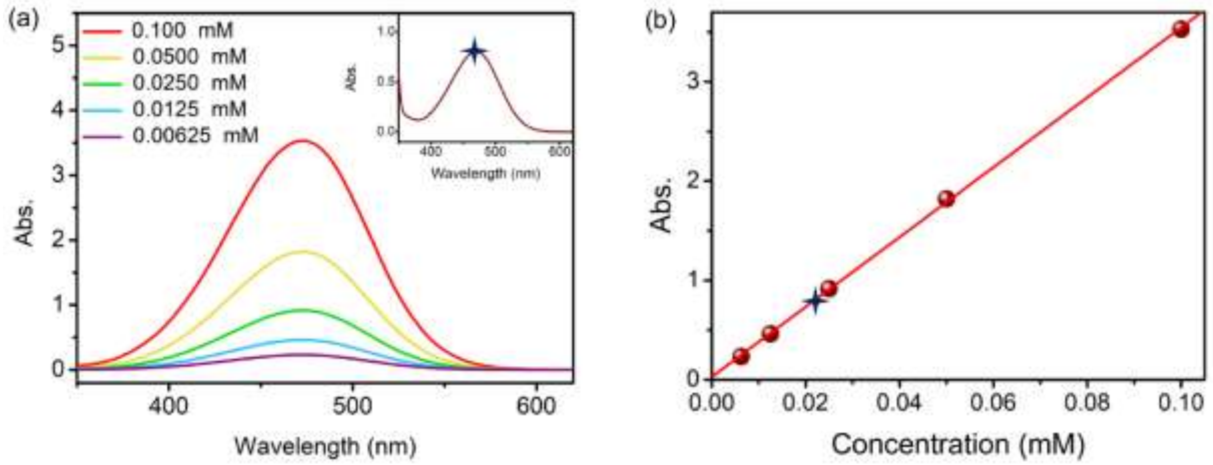

Figure S6.

(a) Absorbance UV-vis spectra of DASE dye dissolved DMF solution with different dye contents. Inset: absorbance UV-vis spectrum of 5.5 mg DASE@ZJU-67 dissolved 40 mL DMF solution (10 μL HCl) with the maximum of 0.805. (b) DASE content dependence of the absorbance peak gives a linear relationship in DMF, showing the as-prepared solution of approximately 0.023 mM.

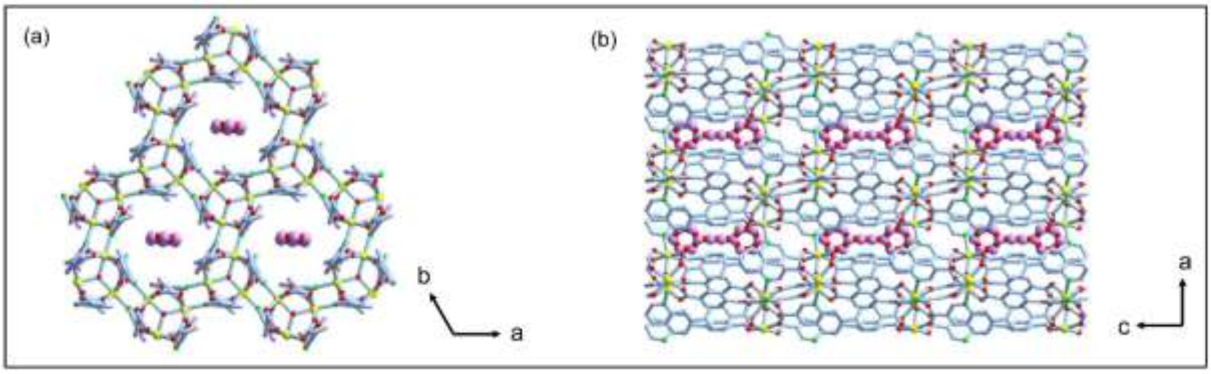

Figure S7.

(a-b) X-Ray single crystal structure of DSAE@ZJU-67 as viewed along *c*-axis (a), *b*-axis (b). Through analyzing the electron density, the linear dye molecules are indeed aligned along 1-D channels of ZJU-67. The molecular shape is similar to the dumbbell. The complete dye molecule structure cannot be obtained. The possible reasons are listed below. Firstly, there is no relatively strong force between dye and MOF. The arrangement of dye molecules is not completely in uniform orientations, which may increase the disordering. Secondly, the ethyl group from the pyridinium cation and the dimethylamine group are flexible, resulting in the disordering of electron density. Thirdly, the electron density should follow the related symmetrical operation of the crystal space group during treating the data collected, which may cause the undesirable symmetry.

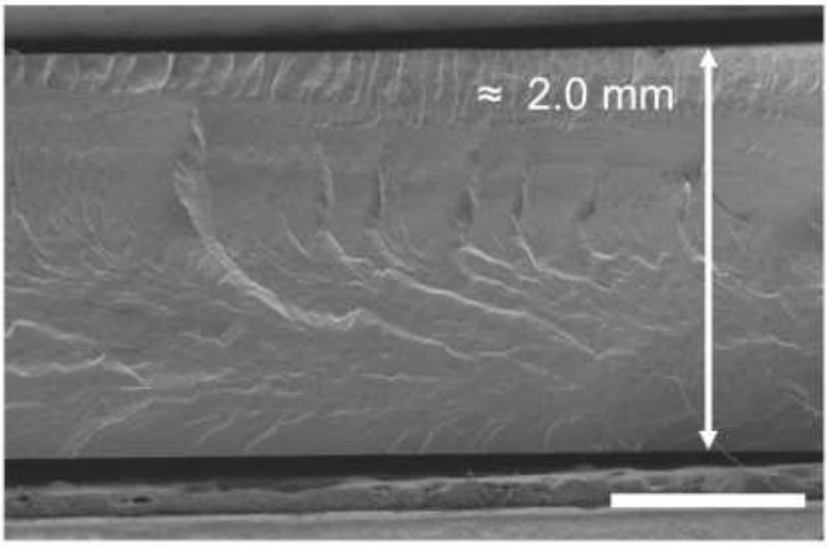

Figure S8.
SEM image of the MMMs brittle fracture surfaces with three layers. Scale bars, 1 mm.

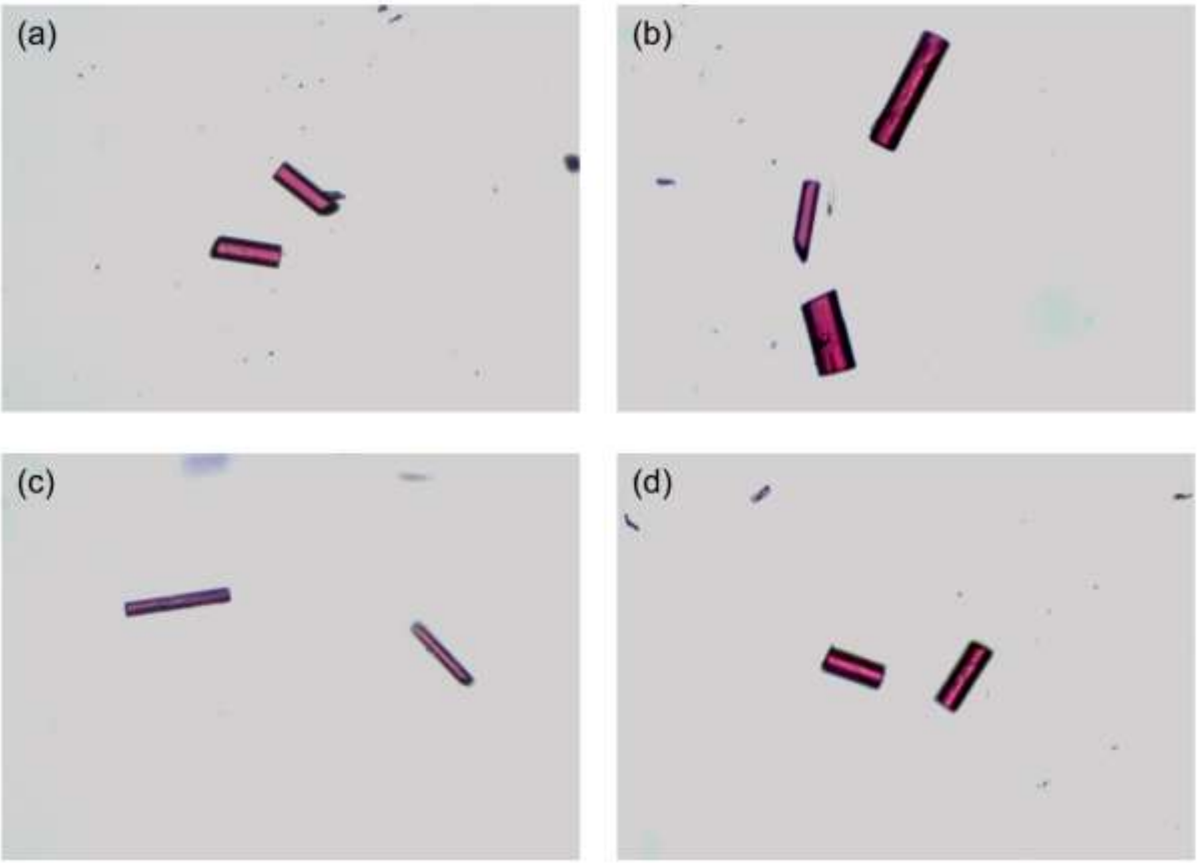

Figure S9.
(a-d) Micrographs from some areas in the MMM with the DASE@ZJU-67 crystals disorder arrangement.

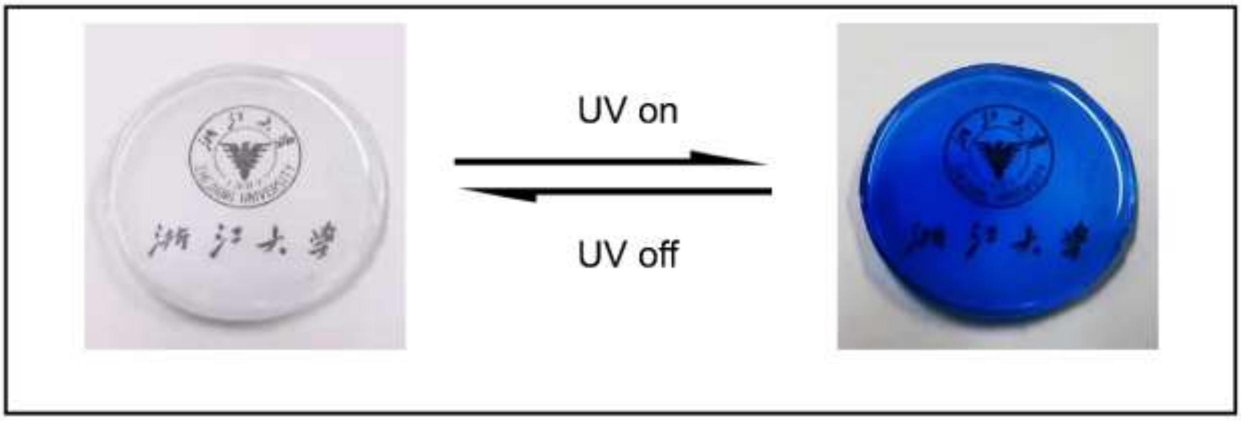

Figure S10.
Color changes of the MOF-based MMM under the switchable UV irradiation.

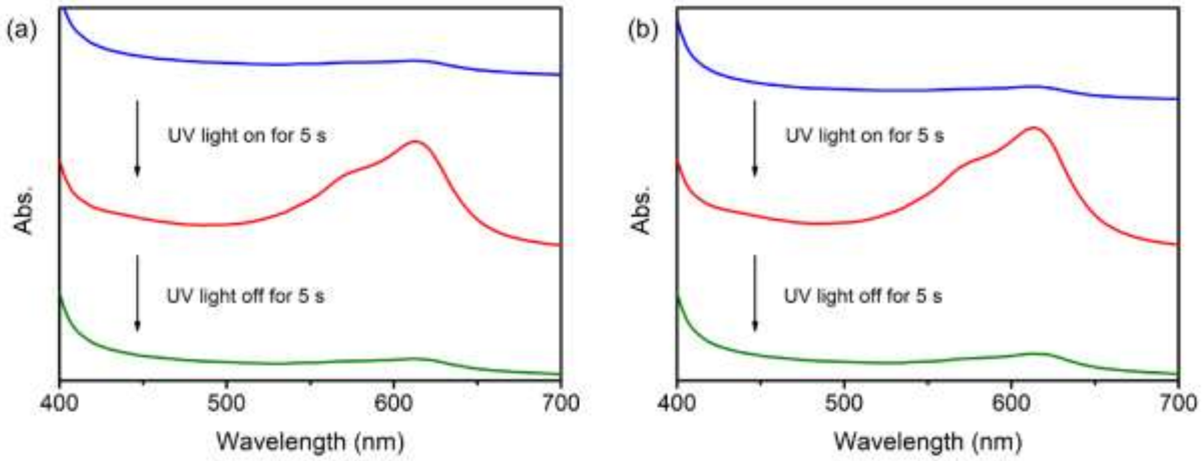

Figure S11.
(a-b) UV-vis absorption spectra of the SO doped PDMS treated under the irradiation of switchable UV light in room light (a) and in dark (b).

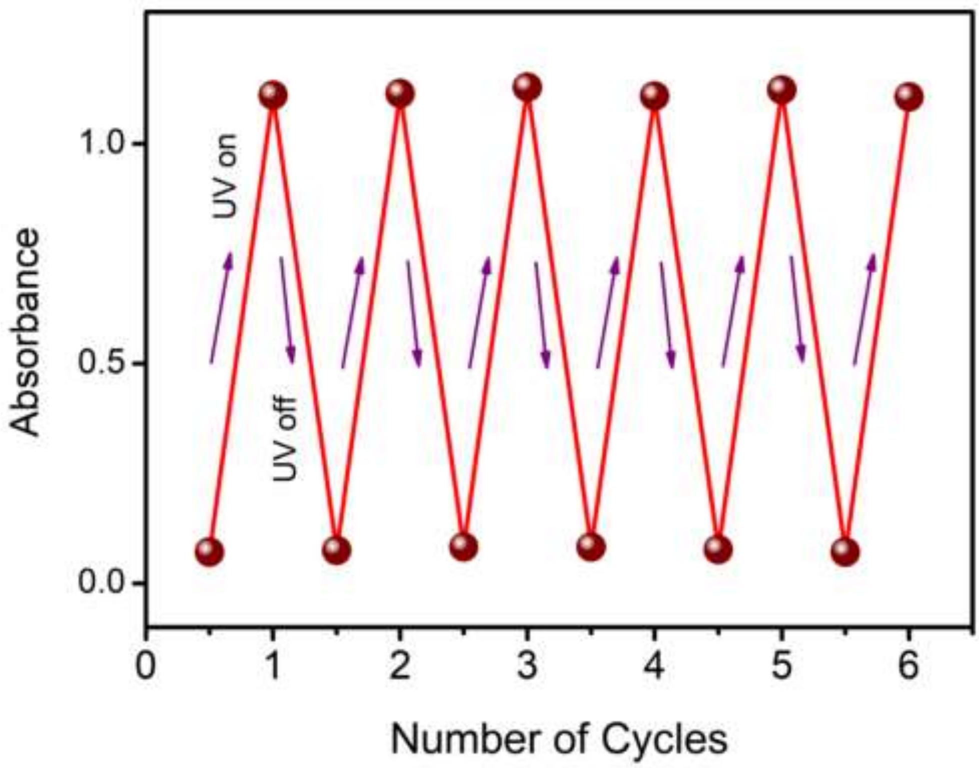

Figure S12.
Switching cycles for the absorbance of the SO doped PDMS under the switchable irradiation of UV light.

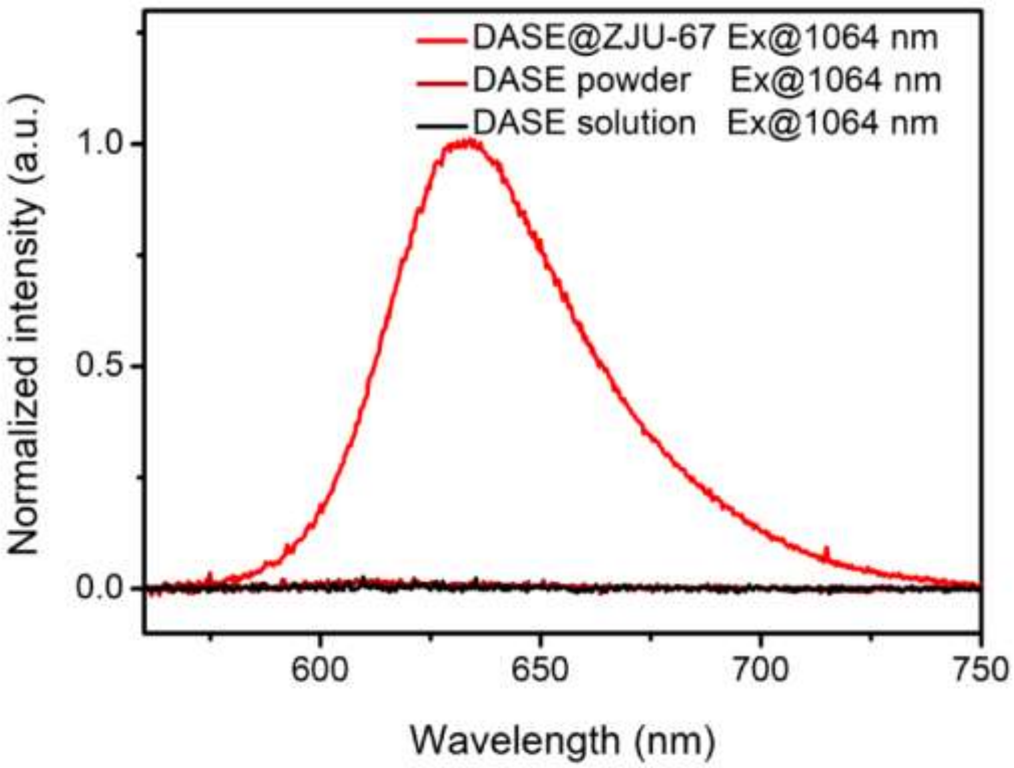

Figure S13.
Emission spectra of DASE dye and DASE@ZJU-67 when excited by 1064 nm fs laser.

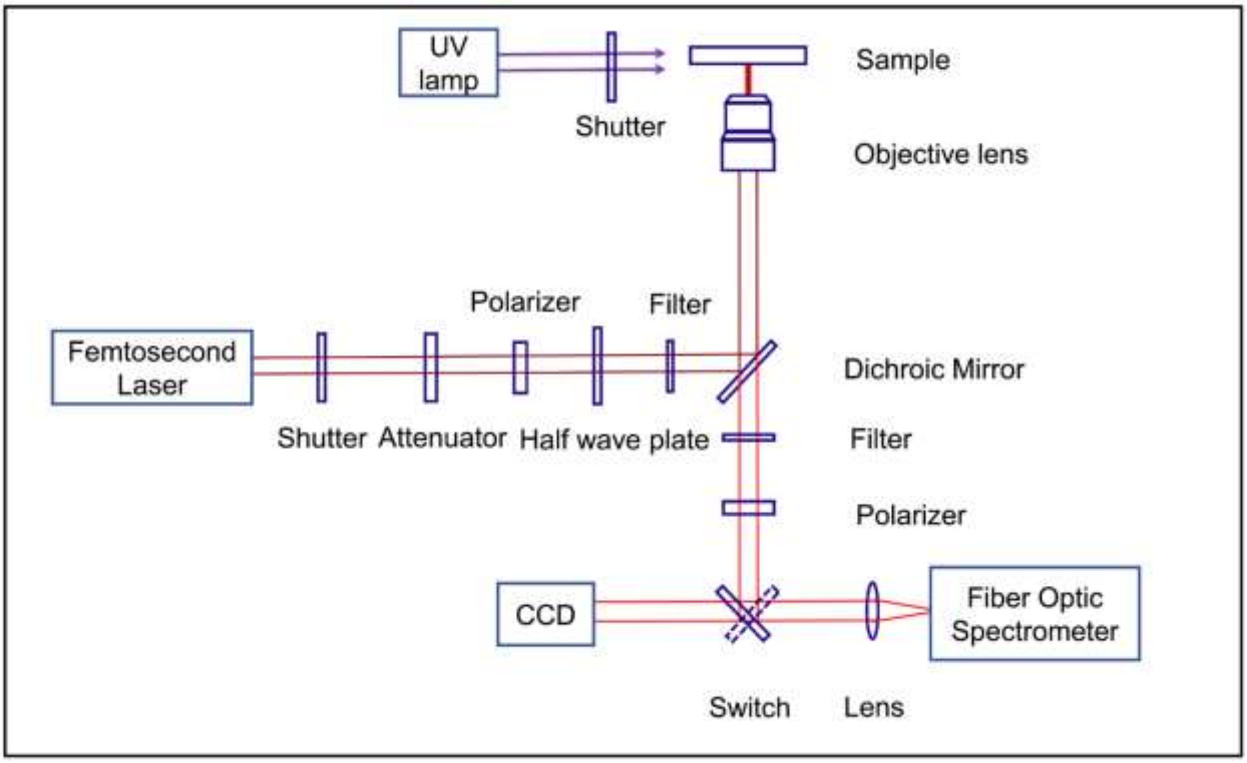

Figure S14.
Schematic diagram of the experimental setup for exploring the up-conversion optical properties.

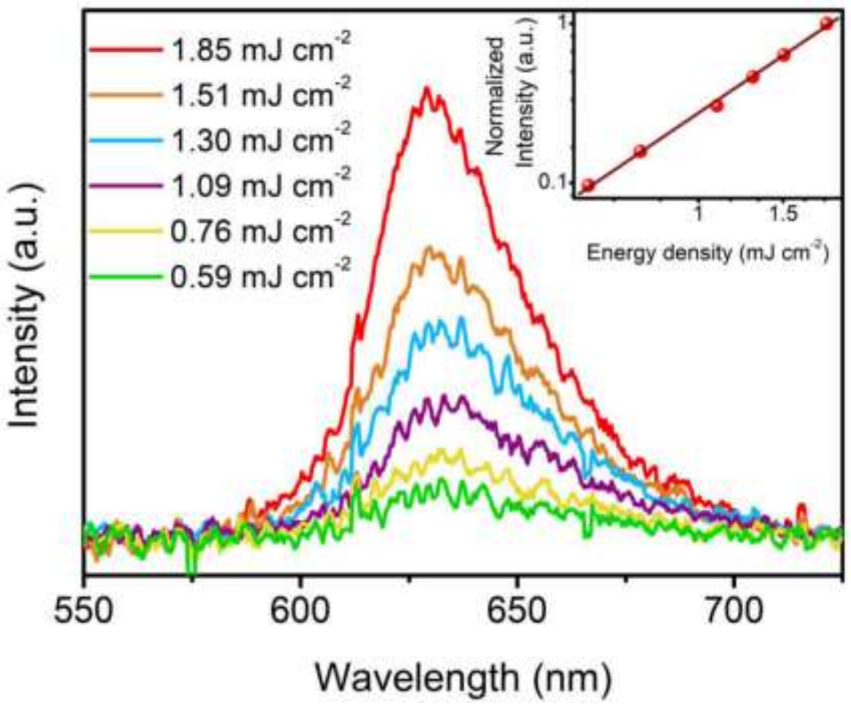

Figure S15.
1064 nm pumped emission spectra of DASE@ZJU-67 microcrystal. Insets: the log-log plots of TPF intensity versus excitation energy density, showing a linear relationship with a slope of ~ 2.03 (≈ 2.00).

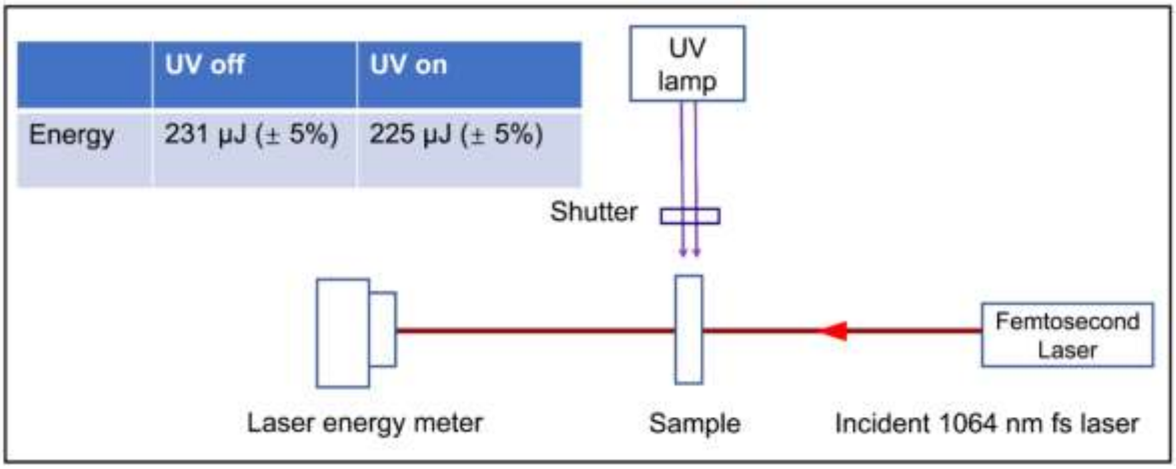

Figure S16.
Pump energy test of laser passing through the as-prepared MMM before and after UV irradiation. When the UV light was off and the photochromic molecule was at SO state, the pump energy was 231 μJ (± 5%). When the UV light was on and the photochromic molecule was at MC state, the pump energy was 225 μJ (± 5%). Such slight change (≈ 2.6%) might be due to the change of refractive index in the MMM or the weak two-photon absorption of MC.

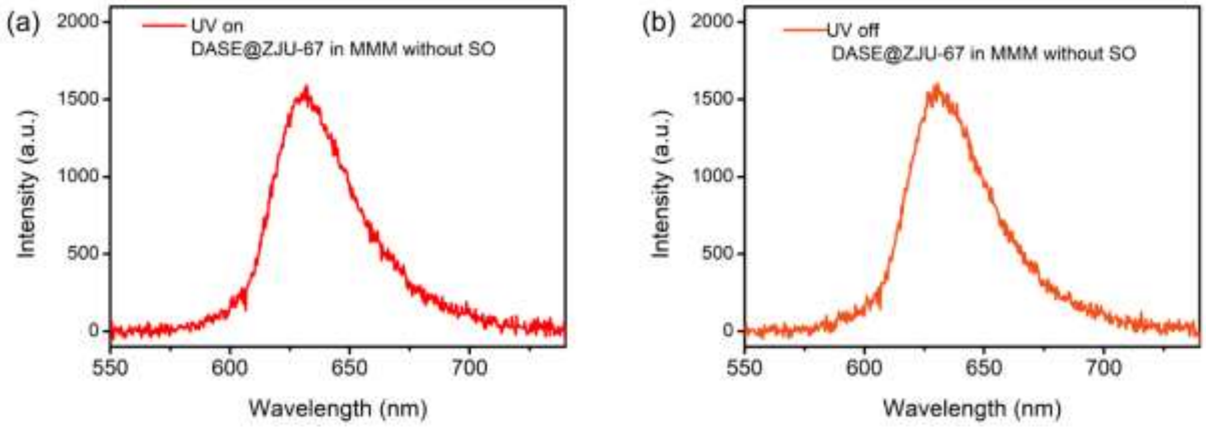

Figure S17.

(a-b) TPF emission intensity of the excited DASE@ZJU-67 in the blank control MMM sample without adding SO, when the UV light is in the on/off states.

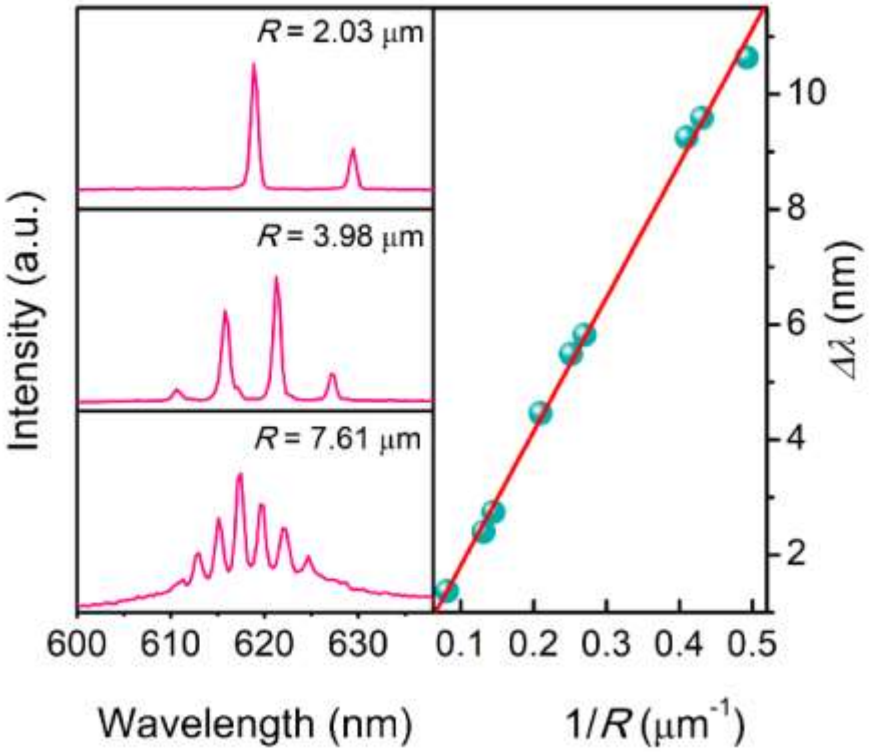

Figure S18.

The lasing spectra of the selected DASE@ZJU-67 microcrystals with different sizes (left) and the linear relationship between Δ*λ* and 1/*R* (right). Δ*λ* is the mode spacing and *R* is the MOF side length.

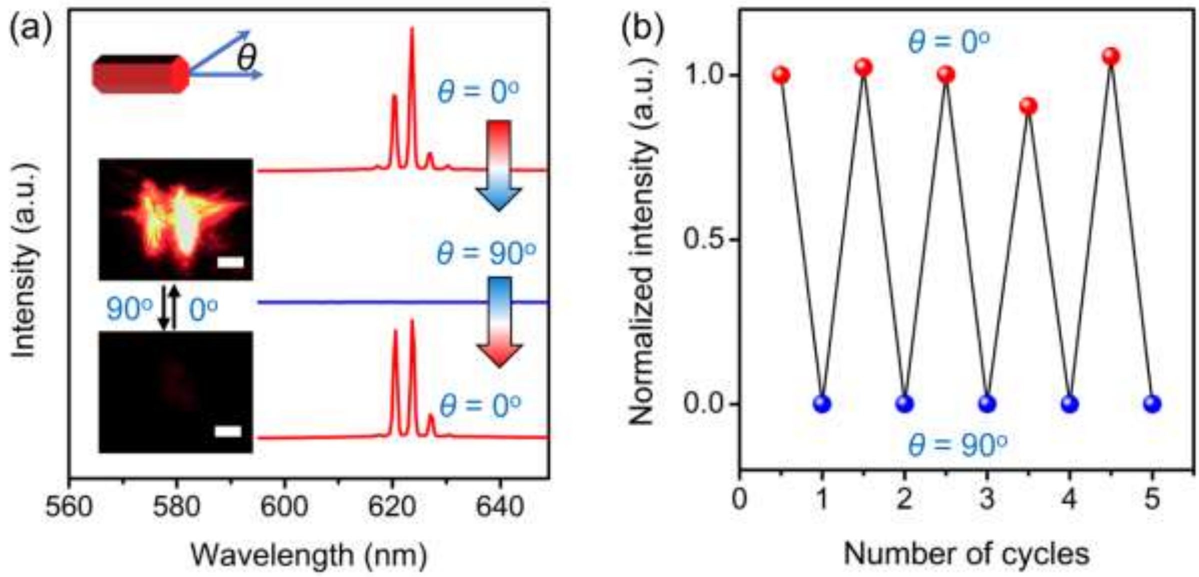

Figure S19.

(a) TPP lasing spectra from the excited DASE@ZJU-67 single crystal when $\theta = 0^o$ or 90° (θ is defined as the angle between the emission-detected polarization direction and MOF longitudinal direction as well as the *c*-axis direction). Insets: micrographs of the excited DASE@ZJU-67 single crystal. Scale bars, 10 μm. (b) Visualization of the switching cycles. The emission intensities change greatly when the emission-detected polarization direction is tuned ($\theta = 0^o$ or 90°).

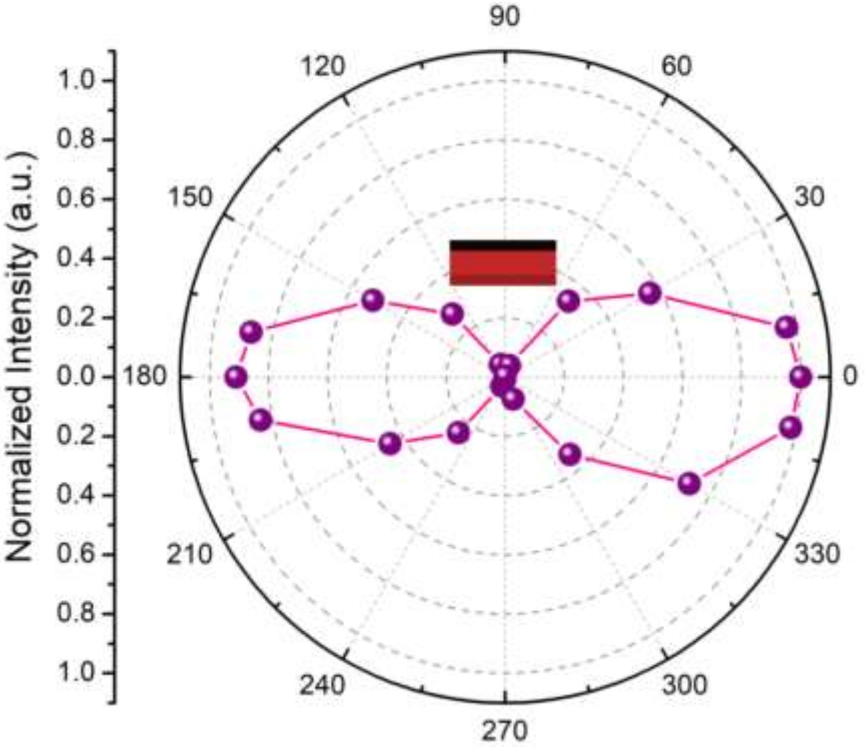

Figure S20.
The as-obtained polar plot by using a 1064 nm polarized fs laser as the excitation source, of which the polarization direction can be changed by rotating a half wave plate. The excited DASE@ZJU-67 reveals an optical anisotropic emission.

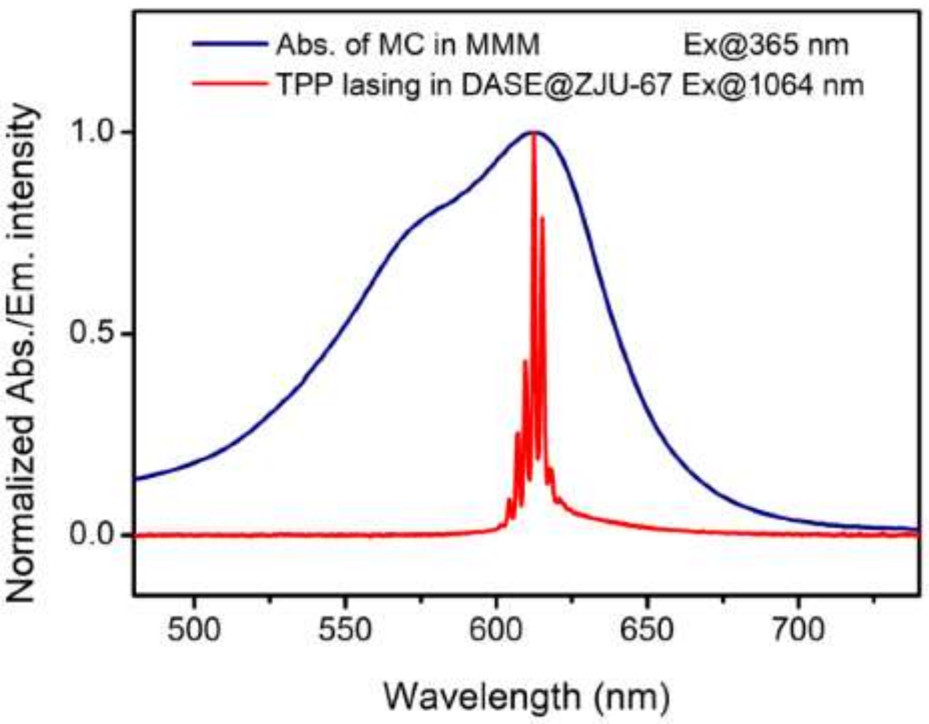

Figure S21.
Absorbance spectrum of MC in MMMs and TPP lasing spectrum of DASE@ZJU-67, showing an obvious overlap.

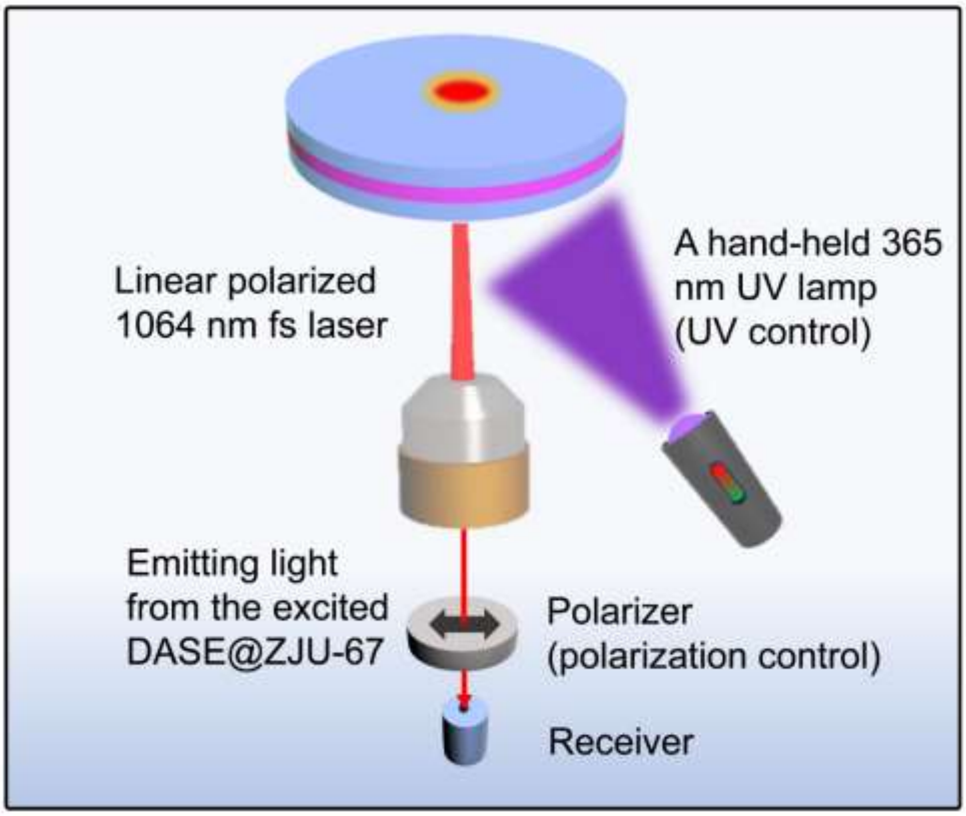

Figure S22.
Schematic diagram of optical switching process based on switchable UV irradiation (on/off states) and tunable emission-detected polarization direction (θ = 0° or 90°). UV control: UV irradiation control; polarization control: emission-detected polarization direction control.

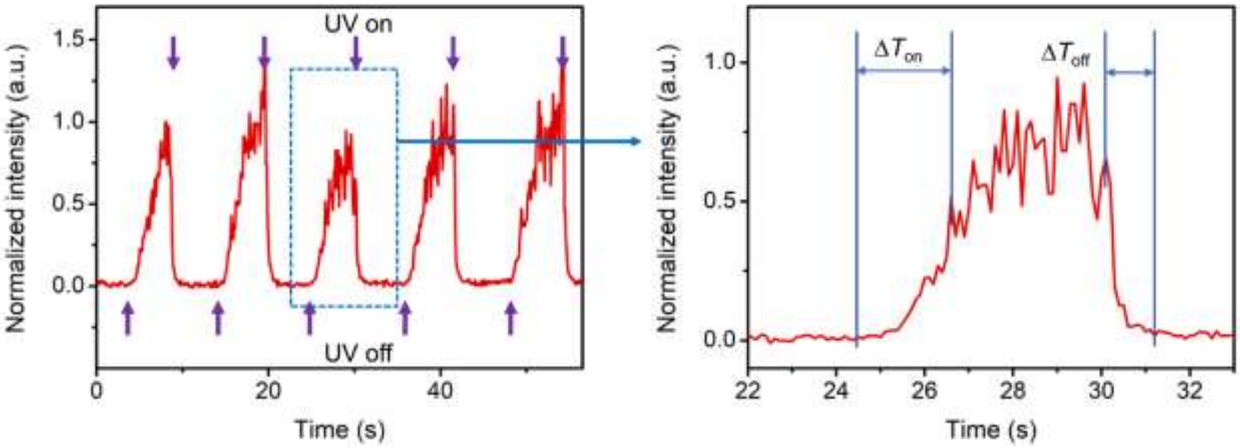

Figure S23.

Switchable TPP lasing via controlling UV light (on/off) for several cycles at room temperature in room light (left) and the third one is selected as an example for further explanation (right). Here, we defined that the time interval from the beginning of the UV light off to the appearance of TPP lasing is the turning on response time (ΔT_{on}); the time interval from the beginning of UV light on to the complete disappearance of TPP lasing is the turning off response time (ΔT_{off}).

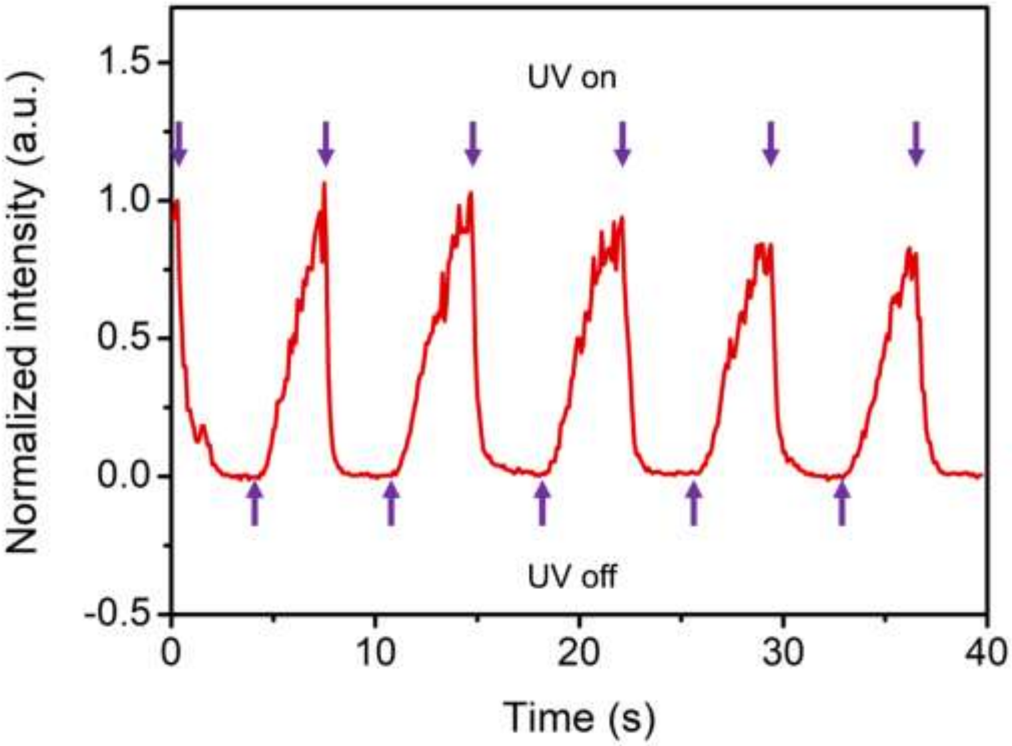

Figure S24.
Switchable TPP lasing via controlling UV light (on/off) for several cycles at room temperature in dark.

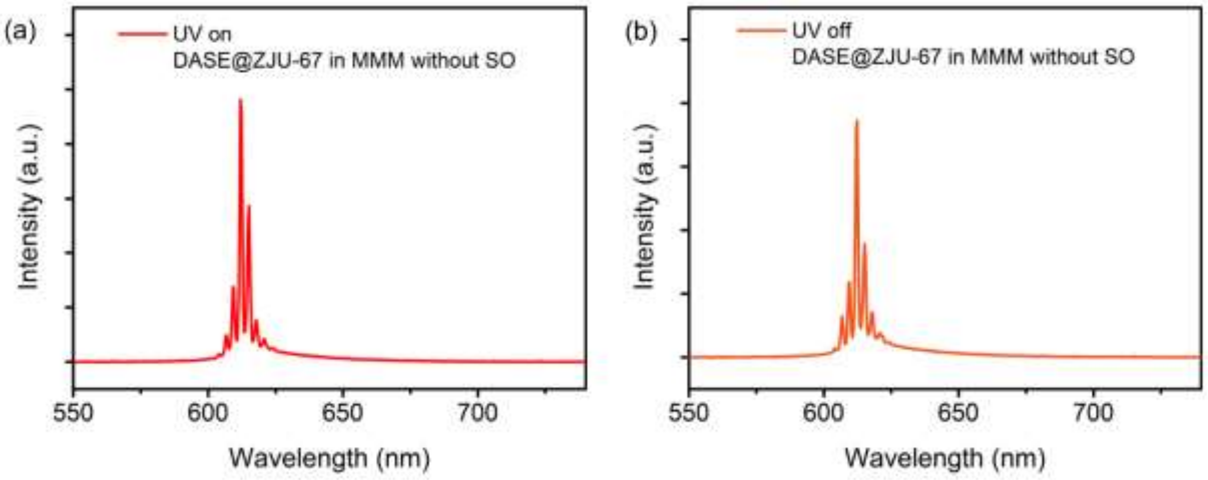

Figure S25.

(a-b) TPP lasing intensity of excited DASE@ZJU-67 in the blank control MMM without adding SO, when the UV light is in the on/off states.

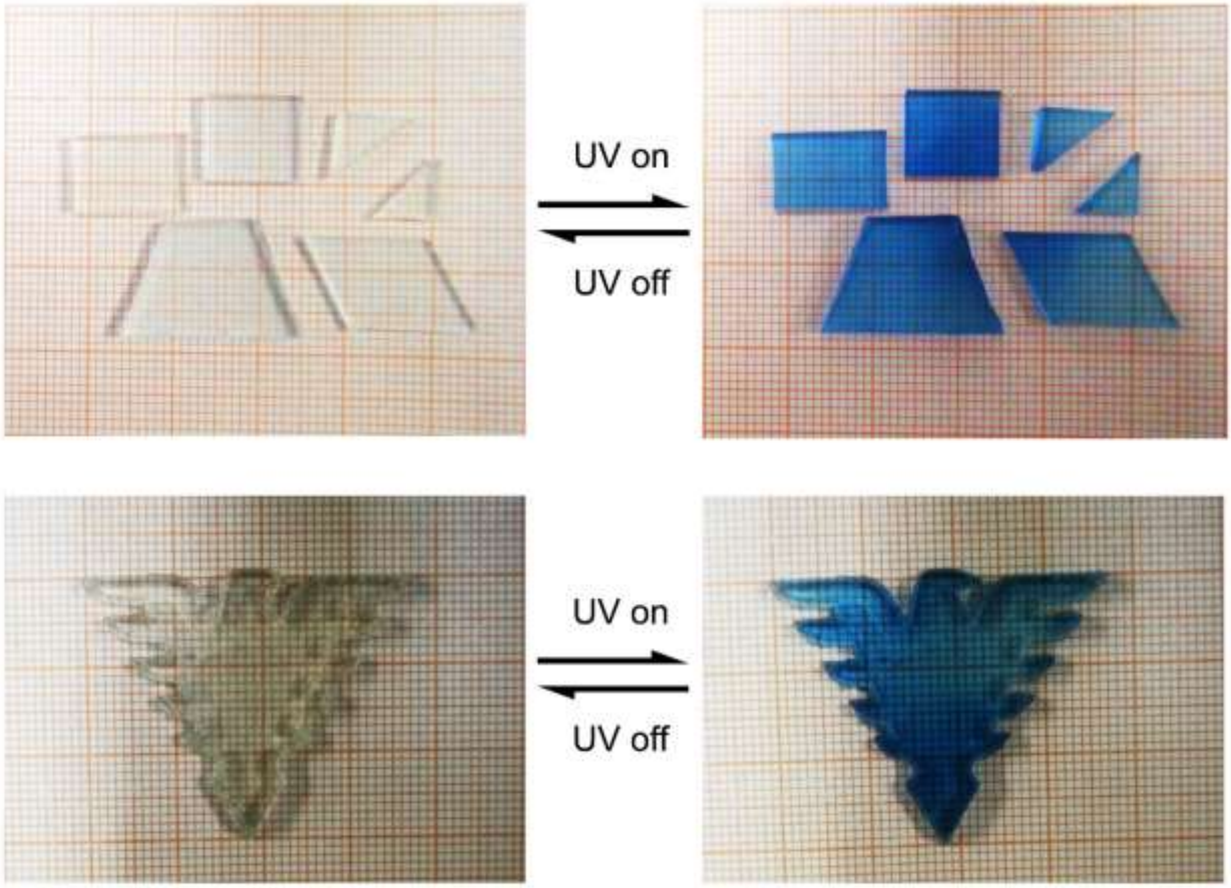

Figure S26.
Tailored or formed MOF-based MMMs with various shapes under the switchable UV light irradiation.

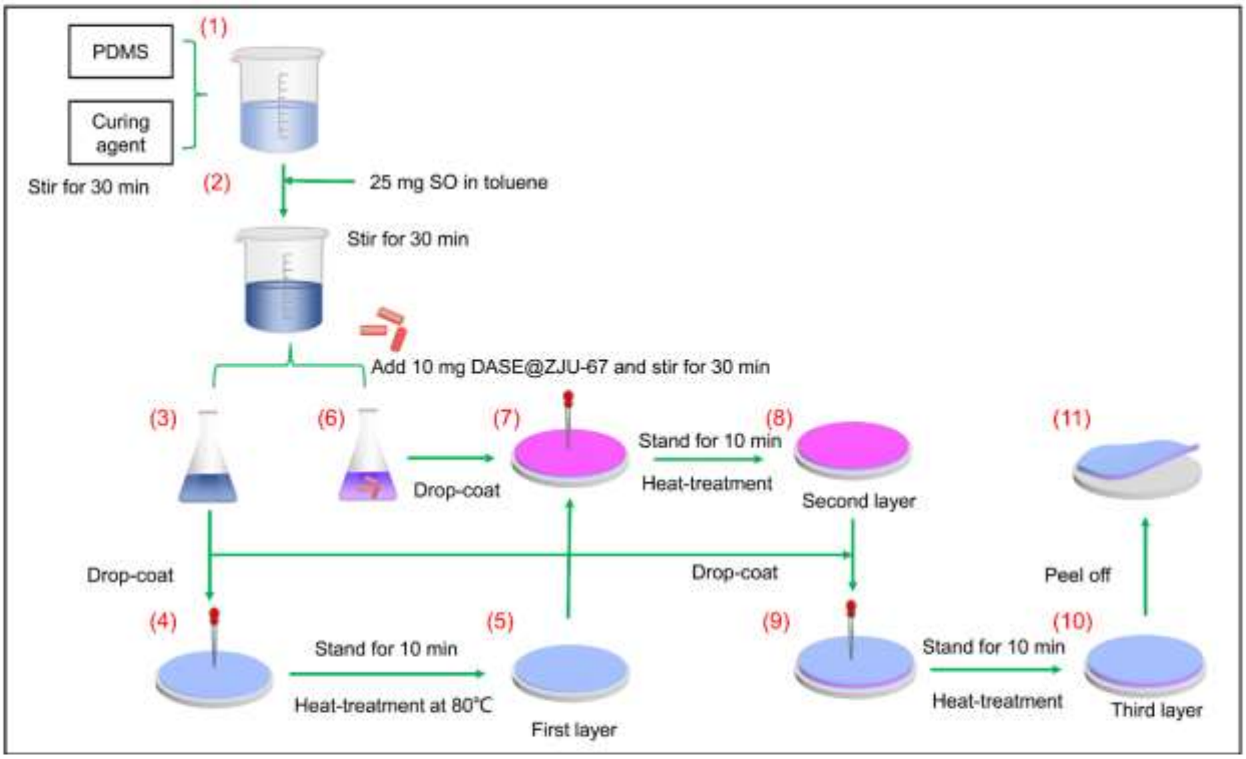

Figure S27.
Schematic diagram of the MOF-based MMM preparation process in details.

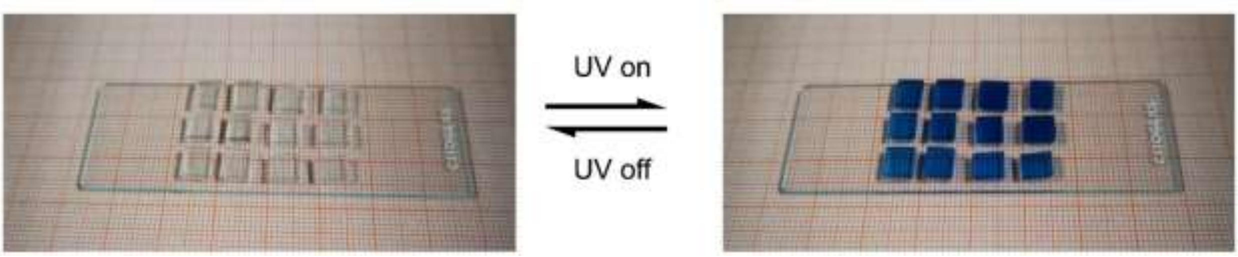

Figure S28.

For the TPP lasing and optical switching tests, the MMMs were tailored and divided into multiple rectangular membranes with the sizes of approximately 5 × 5 mm^2. The as-prepared MMMs were displayed (with and without UV light irradiation).

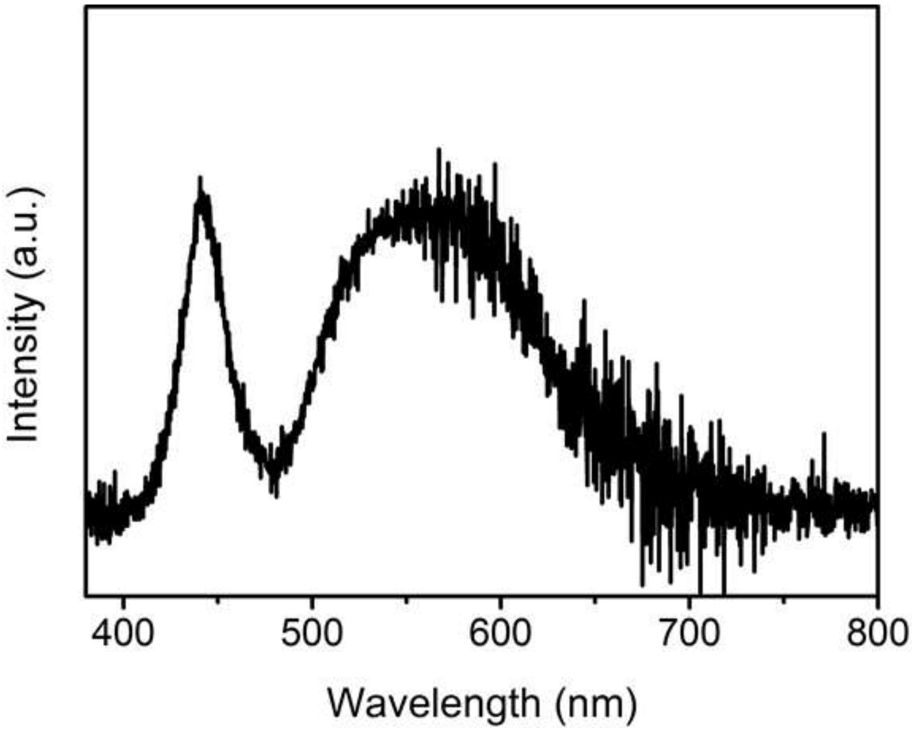

Figure S29.
The spectrum of room light at room temperature in the experimental environment.

References

[1] D. Kottilil, M. Gupta, C. Vijayan, P. K. Bharadwaj, W. Ji, *Adv. Funct. Mater.* **2020**, *30*, 2003294.
[2] M. Gupta, Z. Zhu, D. Kottilil, B. B. Rath, W. Tian, Z. K. Tan, X. Liu, Q. H. Xu, W. Ji, J. J. Vittal, *ACS Appl. Mater. Interfaces* **2021**, *13*, 60163.
[3] A. Alexander, M. Gupta, D. Kottilil, B. B. Rath, J. J. Vittal, W. Ji, *ACS Appl. Mater. Interfaces* **2021**, *13*, 31891.
[4] T. Tian, Y. Wang, W. Zhang, B. Wang, C. Fan, G. You, S. Yuan, G. Xu, M. Li, C. Xu, B. Cai, *ACS Photonics* **2020**, *7*, 2132.
[5] D. C. Mayer, A. Manzi, R. Medishetty, B. Winkler, C. Schneider, G. Kieslich, A. Pothig, J. Feldmann, R. A. Fischer, *J Am Chem Soc* **2019**, *141*, 11594.
[6] Z. Wang, C. Y. Zhu, J. T. Mo, P. Y. Fu, Y. W. Zhao, S. Y. Yin, J. J. Jiang, M. Pan, C. Y. Su, *Angew. Chem. Int. Ed.* **2019**, *58*, 9752.
[7] H. S. Quah, W. Chen, M. K. Schreyer, H. Yang, M. W. Wong, W. Ji, J. J. Vittal, *Nat. Commun.* **2015**, *6*, 7954.
[8] R. Medishetty, J. K. Zareba, D. Mayer, M. Samoc, R. A. Fischer, *Chem. Soc. Rev.* **2017**, *46*, 4976.
[9] L. S. Rohwer, J. E. Martin, *J. Lumin.* **2005**, *115*, 77.
[10] N. S. Makarov, M. Drobizhev, A. Rebane, *Opt. Express* **2008**, *16*, 4029.
[11] H. He, Y. Cui, B. Li, B. Wang, C. Jin, J. Yu, L. Yao, Y. Yang, B. Chen, G. Qian, *Adv. Mater.* **2019**, *31*, e1806897.
[12] Y. F. Zhou, S. Y. Feng, X. M. Wang, *J. Mol. Struct.* **2002**, *613*, 91.
[13] J. Armitage, V. M. Yashchuk, V. Y. Kudrya, M. Y. Losytskyy, V. P. Tokar, S. M. Yarmoluk, I. M. Dmytruk, V. M. Prokopets, V. B. Kovalska, A. O. Balanda, D. V. Kryvorotenko, T. Y. Ogul'chansky, *Proc. SPIE-Int. Soc.* **2007**, *6796*, 67960M.
[14] G. S. He, L. S. Tan, Q. Zheng, P. N. Prasad, *Chem. Rev.* **2008**, *108*, 1245.
[15] Z. Li, G. Wang, Y. Ye, B. Li, H. Li, B. Chen, *Angew. Chem. Int. Ed.* **2019**, *58*, 18025.
[16] M. Tu, H. Reinsch, S. Rodriguez-Hermida, R. Verbeke, T. Stassin, W. Egger, M. Dickmann, B. Dieu, J. Hofkens, I. F. J. Vankelecom, N. Stock, R. Ameloot, *Angew. Chem. Int. Ed.* **2019**, *58*, 2423.
[17] N. K. Kulachenkov, D. Sun, Y. A. Mezenov, A. N. Yankin, S. Rzhevskiy, V. Dyachuk, A. Nomine, G. Medjahdi, E. A. Pidko, V. A. Milichko, *Angew. Chem. Int. Ed.* **2020**, *59*, 15522.
[18] D. E. Williams, J. A. Rietman, J. M. Maier, R. Tan, A. B. Greytak, M. D. Smith, J. A. Krause, N. B. Shustova, *J. Am. Chem. Soc.* **2014**, *136*, 11886.
[19] K. Muller, J. Wadhwa, J. Singh Malhi, L. Schottner, A. Welle, H. Schwartz, D. Hermann, U. Ruschewitz, L. Heinke, *Chem. Commun.* **2017**, *53*, 8070.
[20] B. J. Furlong, M. J. Katz, *J. Am. Chem. Soc.* **2017**, *139*, 13280.
[21] I. Mjejri, C. M. Doherty, M. Rubio-Martinez, G. L. Drisko, A. Rougier, *ACS Appl. Mater. Interfaces* **2017**, *9*, 39930.
[22] K. AlKaabi, Casey R. Wade, M. Dincă, *Chem* **2016**, *1*, 264.
[23] J. Liu, X. Y. Daphne Ma, Z. Wang, L. Xu, T. Xu, C. He, F. Wang, X. Lu, *ACS Appl. Mater. Interfaces* **2020**, *12*, 7442.
[24] R. Li, S. Li, Q. Zhang, Y. Li, H. Wang, *Inorg. Chem. Commun.* **2021**, *123*, 108354.
[25] W.-F. Chen, B.-W. Liu, S.-M. Pei, Q.-N. Yan, X.-M. Jiang, G.-C. Guo, *Chem. Mater.* **2021**, *33*, 3729.
[26] W.-Y. Zhang, Q. Ye, D.-W. Fu, R.-G. Xiong, *Adv. Funct. Mater.* **2017**, *27*, 1603945.
[27] Z. Sun, T. Chen, X. Liu, M. Hong, J. Luo, *J. Am. Chem. Soc.* **2015**, *137*, 15660.
[28] S. Y. Zhang, X. Shu, Y. Zeng, Q. Y. Liu, Z. Y. Du, C. T. He, W. X. Zhang, X. M. Chen, *Nat. Commun.* **2020**, *11*, 2752.
[29] C. Sun, C. Yang, P. X. Li, M. S. Wang, G. C. Guo, *Inorg. Chem.* **2021**, *60*, 9278.

[30] L. He, P.-P. Shi, M.-M. Zhao, C.-M. Liu, W. Zhang, Q. Ye, *Chem. Mater.* **2021**, *33*, 799.
[31] X. Liu, C. Ji, Z. Wu, L. Li, S. Han, Y. Wang, Z. Sun, J. Luo, *Chem. Eur. J.* **2019**, *25*, 2610.
[32] L. Zhou, X. Zheng, P. P. Shi, Z. Zafar, H. Y. Ye, D. W. Fu, Q. Ye, *Inorg. Chem.* **2017**, *56*, 3238.
[33] P. Serra-Crespo, M. A. van der Veen, E. Gobechiya, K. Houthoofd, Y. Filinchuk, C. E. Kirschhock, J. A. Martens, B. F. Sels, D. E. De Vos, F. Kapteijn, J. Gascon, *J. Am. Chem. Soc.* **2012**, *134*, 8314.
[34] Z. Wu, X. Liu, C. Ji, L. Li, S. Wang, Z. Sun, W. Zhang, Y. Peng, J. Luo, *J. Mater. Chem. C* **2018**, *6*, 9532.
[35] Z. Sun, J. Luo, S. Zhang, C. Ji, L. Zhou, S. Li, F. Deng, M. Hong, *Adv. Mater.* **2013**, *25*, 4159.
[36] Y. Zeng, C. L. Hu, W. J. Xu, T. W. Zeng, Z. X. Zhu, X. X. Chen, D. X. Liu, Y. J. Chen, Y. B. Zhang, W. X. Zhang, X. M. Chen, *Angew. Chem. Int. Ed.* **2022**, *61*, e202110082.
[37] T. Chen, Z. Sun, S. Zhao, C. Ji, J. Luo, *J. Mater. Chem. C* **2016**, *4*, 266.
[38] S.-L. Li, H.-M. Xu, Y. Zhang, W. Yang, Z. Qi, J. Zhang, X.-M. Zhang, *Cryst. Growth Des.* **2021**, *21*, 5752.
[39] K. Tao, Z. Wu, S. Han, J. Zhang, C. Ji, Y. Wang, W. Zhang, J. Luo, Z. Sun, *J. Mater. Chem. C* **2018**, *6*, 4150.
[40] A. Priimagi, K. Ogawa, M. Virkki, J. Mamiya, M. Kauranen, A. Shishido, *Adv. Mater.* **2012**, *24*, 6410.
[41] J. Zhang, S. Han, X. Liu, Z. Wu, C. Ji, Z. Sun, J. Luo, *Chem. Commun.* **2018**, *54*, 5614.
[42] Z. Sun, S. Li, S. Zhang, F. Deng, M. Hong, J. Luo, *Adv. Opt. Mater.* **2014**, *2*, 1199.
[43] C. Ji, Z. Sun, S. Zhang, S. Zhao, T. Chen, Y. Tang, J. Luo, *Chem. Commun.* **2015**, *51*, 2298.
[44] C. Y. Pan, X. R. Yang, L. Xiong, Z. W. Lu, B. Y. Zhen, X. Sui, X. B. Deng, L. Chen, L. M. Wu, *J. Am. Chem. Soc.* **2020**, *142*, 6423.
[45] J. Boixel, V. Guerchais, H. Le Bozec, D. Jacquemin, A. Amar, A. Boucekkine, A. Colombo, C. Dragonetti, D. Marinotto, D. Roberto, S. Righetto, R. De Angelis, *J. Am. Chem. Soc.* **2014**, *136*, 5367.
[46] P. X. Li, M. S. Wang, M. J. Zhang, C. S. Lin, L. Z. Cai, S. P. Guo, G. C. Guo, *Angew. Chem. Int. Ed.* **2014**, *53*, 11529.
